# Ambiguity in logic-based models of gene regulatory networks: An integrative multi-perturbation analysis

**DOI:** 10.1371/journal.pone.0206976

**Published:** 2018-11-20

**Authors:** Amir Reza Alizad-Rahvar, Mehdi Sadeghi

**Affiliations:** 1 School of Biological Sciences, Institute for Research in Fundamental Sciences (IPM), Tehran, Iran; 2 National Institute for Genetic Engineering and Biotechnology, Tehran, Iran; Universite de Nantes, FRANCE

## Abstract

Most studies of gene regulatory network (GRN) inference have focused extensively on identifying the interaction map of the GRNs. However, in order to predict the cellular behavior, modeling the GRN in terms of logic circuits, i.e., Boolean networks, is necessary. The perturbation techniques, e.g., knock-down and over-expression, should be utilized for identifying the underlying logic behind the interactions. However, we will show that by using only transcriptomic data obtained by single-perturbation experiments, we cannot observe all regulatory interactions, and this invisibility causes ambiguity in our model. Consequently, we need to employ the data of multiple omics layers (genome, transcriptome, and proteome) as well as multiple perturbation experiments to reduce or eliminate ambiguity in our modeling. In this paper, we introduce a multi-step perturbation experiment to deal with ambiguity. Moreover, we perform a thorough analysis to investigate which types of perturbations and omics layers play the most important role in the unambiguous modeling of the GRNs and how much ambiguity will be eliminated by considering more perturbations and more omics layers. Our analysis shows that performing both knock-down and over-expression is necessary in order to achieve the least ambiguous model. Moreover, the more steps of the perturbation are taken, the more ambiguity is eliminated. In addition, we can even achieve an unambiguous model of the GRN by using multi-step perturbation and integrating transcriptomic, protein-protein interaction, and *cis*-element data. Finally, we demonstrate the effect of utilizing different types of perturbation experiment and integrating multi-omics data on identifying the logic behind the regulatory interactions in a synthetic GRN. In conclusion, relying on the results of only knock-down experiments and not including as many omics layers as possible in the GRN inference, makes the results ambiguous, unreliable, and less accurate.

## Introduction

Owing to the development of high-throughput measurement technologies, such as the DNA microarray and ChIP-on-chip technique, molecular biology relies increasingly on mathematics, physics, engineering, and computing science for analyzing the quantitative data and modeling of biological networks. Hence, a new emerging discipline called *computational systems biology* is attempting to discover the cellular networks by using computational methods.

Some of the most important biological networks, whose identification helps us to understand the biological function of the cells and also has immediate applications such as cancer prediction and drug development, are the *gene regulatory networks* (GRNs) [1]. Indeed, the GRN models provide information about the pathway to which a gene belongs and the underlying regulatory interactions. Furthermore, GRNs can introduce potential drug targets by finding the pathway initiators. The ultimate goal of the GRN inference is to demonstrate and predict the behavior of the cell in response to different environmental and developmental signals and stimuli. Gene regulatory interactions in the cell can change the expression level of the genes, resulting in changes in the amount of the gene products, i.e., RNA and protein. These regulatory interactions make up a vast number of the GRNs consisting of the regulated genes and the regulatory factors (RFs), e.g., transcription factors (TFs), miRNAs, histone acetyltransferases and deacetylases, and kinases. A regulated gene in a GRN could be itself the regulator of other genes as well.

### Modeling of the GRN

The complexity of the behavior of the molecules and biochemical species in the cell makes the task of the GRN modeling very challenging. A number of computational approaches have been proposed for GRN inference, for example, differential equations, Boolean networks, Bayesian networks, and information theoretical approaches [2]. To simplify the task of the network inference and have a non-parametric model of the GRN, we can utilize a binary variable. This variable can take only two states, say 0 (OFF) and 1 (ON). For example, in the process of the gene regulation at the transcription level, there is a sigmoidal relationship between the concentration of the mRNA of the regulatory gene and that of the target gene [3]. This relationship can be approximated as a Boolean switch having two states ON (saturated) and OFF (non-saturated). A regulator gene is OFF while the concentration of the corresponding mRNA is not high enough to regulate the target gene. On the other hand, the ON state of the regulator means that the concentration of its mRNA has reached a threshold of the functional activity.

By considering a gene as a binary variable, the logic-based models can be used to model the gene regulation. In this paper, the term *logic* only refers to Boolean logic, e.g., logic gates and logic functions. Indeed, a logic circuit consisting of the logic gates can represent a regulatory interaction whose inputs are the regulators, e.g., transcription factors, and its single output is the target gene. In numerous cases, the regulation process can be modeled by using a single logic gate. However, we need to use more complex circuits made by multiple gate combinations for more complicated regulations. The logic circuit is the electronic implementation of the Boolean function *f* that maps the current state of the regulators (inputs) to the outcome state of the target gene (output). All Boolean functions can be described by the combination of three basic logical operators: AND, OR, and NOT [4], denoted by AB (or A•B), A+B, and $\bar{A}$, respectively, for two Boolean variables A and B. The OR logic gate indicates that the presence of either regulator is enough for the expression of the target gene. In contrast, the AND logic gate means that all RFs are necessary to express the regulated gene. There are 2^*k*^ possible combinations of the input states (combinations of 0s and 1s) for *k* input variables of *f*. Accordingly, $2^{2^{k}}$ different Boolean functions can be constructed for different combinations of the input states. Moreover, each Boolean function can be represented by a truth table showing the output of *f* corresponding to each of 2^*k*^ possible combinations of its arguments [4]. Although the observation of all input combinations of *f* can result in its unique identification, by utilizing today’s technologies, doing so is either unfeasible or time-consuming and costly for large numbers of *k*. Therefore, it is required to find some solutions by working with only some combinations of the input states. This partial observation of the input combinations is one of the main sources of the ambiguity in GRN inference.

Regardless of what method is utilized for modeling the GRN, the ultimate network of the interacting components can be represented as a graph. To have an implicit indication of causality in the network, we can demonstrate the GRN as a directed graph *G* in two ways [5, 6]:

**Interactive network:** This kind of network denoted by *G*(*V,E*) consists of a set of nodes *V* = {*v*_1_, …, *v*_*n*_} representing *n* genes and a list of directed edges *E* where *v*_*i*_ → *v*_*j*_ and *v*_*i*_ ⊣ *v*_*j*_ show the activatory (up-regulation) and inhibitory (down-regulation) effect of the regulator gene *v*_*i*_ on the regulated gene *v*_*j*_, respectively.
**Boolean network:** The Boolean networks were proposed for the first time by Kauffman as a qualitative logic-based model of the GRNs [3, 7]. The Boolean network denoted by *G*(*V,F*) is a directed graph consisting of a set of nodes *V* and a list of Boolean functions *F* = {*f*_1_, …, *f*_*n*_}. The *i*-th node with indegree of *k* has the Boolean function $f_{i}\left(v_{i_{1}},...,v_{i_{k}}\right)$ meaning that the gene *v*_*i*_ is regulated by *k* genes $v_{i_{1}},...,v_{i_{k}}$ with the underlying logic function *f*_*i*_.

**Fig 1:**
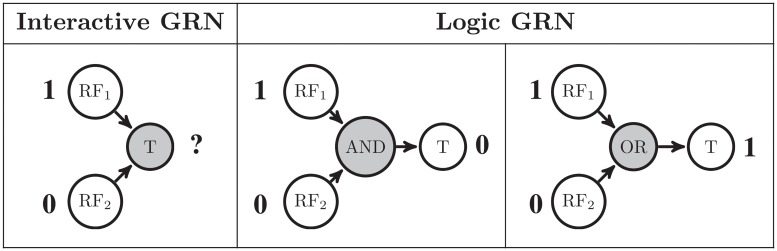
Interactive vs. predictive network. Two regulatory factors RF_1_ and RF_2_ are the activators of the target gene T. By knowing only the steady state of the regulators, we cannot predict the steady state of T based on the interactive GRN model. In contrast, the outcome of the interactions is predictable by knowing the underlying logic behind the interactions in the logic-based GRN model.

Interactive networks merely display the structure of the network as well as the nature of each regulatory interaction, i.e., activation or inhibition. This kind of network cannot predict the behavior of the GRN or the next state of the genes based on their current state [6]. For example, consider two regulatory factors RF_1_ and RF_2_ activating the target gene T with the interactive network RF_1_→T←RF_2_ (Fig 1). Assume that the underlying logic of these interactions is an AND gate and that the steady state of RF_1_ and RF_2_ is 1 and 0, respectively. Consequently, in this case, the state of T would be 0. Now assume that RF_1_ and RF_2_ regulate the target gene as an OR gate; then the output will be 1. Hence, we can get different outputs for the same structure of the network and input states. However, this behavior is not predictable by the interactive network; therefore, we need to know the underlying logic of the network for prediction. In conclusion, a GRN graph that is not a logic-based model cannot achieve most of the goals of the GRN inference. In other words, the lack of the logic behind the network deprives us of an accurate understanding of the behavior of the GRN, which is the main goal of modeling the network. Hence, in our study, we focused on the logic-based modeling of the GRN.

### Perturbation experiment

Traditionally, knock-down or knock-out techniques are usually applied as a perturbation to a group of RFs individually to identify their potential regulated genes and discover the causal effect of the regulators on the target genes (knock-out technique will not be mentioned hereafter). In other words, the gene encoding the intended RF, e.g., the transcription factor, is switched off by knocking-down. Consequently, the corresponding RF degrades due to the lack of the expression of the encoding gene.

Despite the effectiveness of the knock-down-technique in identifying the GRNs, it is useful only for the regulatory genes whose initial (wild-type) state is 1 and we can see their effect by turning them off. For the regulators whose initial state is 0, we can use the over-expression technique as the perturbation method to switch them on and guarantee the existence of the corresponding RFs [8]. Accordingly, to design a comprehensive perturbation experiment, we should first observe the initial unperturbed (wild-type) state of the inputs and outputs. Afterwards, each input state should be altered to its reverse state; i.e., if the steady state of a regulatory gene is 0 (1), we should use the over-expression (knock-down) technique to alter its state. Then, we look at the changes in the output states and recognize a gene as the target of the perturbed RF if the gene’s state is different before and after the perturbation.

Most studies in the literature deploy the perturbation of a single gene at a time to infer the GRNs. The basic assumption in perturbation experiments is that if an RF regulates a gene, the RF’s effect on a target gene or the corresponding phenotypic effects will be inevitably visible after perturbation; hence, if we do not see any change in the state of a gene or its phenotypic effect, we exclude it from the list of the target genes. However, this assumption is not valid for all cases. For example, the existence of duplicates, alternative pathways, and functional overlaps results in little phenotypic effects in many cases [9]. As another example, consider the simple network depicted in Fig 1 with the OR logic gate. Assume that the initial state of both RFs is 1, and consequently, that the output state is also 1. In this case, if the state of either RF_1_ or RF_2_ is changed to 0, the change does not have any effect on the state of the target gene and still remains 1. However, if we perturb both regulators simultaneously and change them to 0, we can see the change in the target gene. Therefore, we are dealing with the *visibility problem* that the effect of some RFs on the target gene is not visible by the single-perturbation experiments. Hence, they can lead in some missing edges in the GRN graph due to the invisibility of some regulatory interactions. Furthermore, if, like many studies in the literature, we use only the knock-down technique as the perturbation, i.e., do not use over-expression, this invisibility becomes even more severe, and consequently, we will have more missing edges in the network.

One approach to deal with the visibility problem is multi-perturbation studies that are essential for the GRN inference [10, 11]. Most of the current studies, however, are still performing single-perturbation techniques because multi-perturbation studies are expensive, time-consuming, and, in some cases, are even biologically or technologically unfeasible. Therefore, we need more advances in technology to make these studies more feasible, time- and cost-efficient than they are currently, in order to perform these studies widely. Until such advances are available, we should be cautious about the potentially misleading results obtained from single-perturbation studies and interpret them carefully.

### Multi-omics data integration

Gene regulation happens in different levels such as in chromatin domains, transcription, post-transcription, translation, and post-translation [12–16]. However, in the vast majority of the studies in the literature, only the transcription data, consisting of the expression level of the genes usually measured by microarray experiments or RNA-seq to reveal the mRNA concentration, is used to infer the GRNs. The reason for the vast usage of this kind of data is the cost-effectiveness, time efficiency, the huge amount of publicly available data resources, and availability of expertise to collect and analyze data. Obviously, each kind of data presents a limited view of the system, and to be able to obtain a more complete picture of the gene regulation, and consequently the complete graph of the GRN, we need to observe the regulatory interactions in all different levels; otherwise, the outcome of the GRN inference will be spurious. Hence, there is an urgent need to collect data from different omics layers, i.e., the genome, transcriptome, proteome, and metabolome layers, and to utilize these heterogeneous data in integrative network inference methods [17–19]. The lack of data in each layer results in the ambiguity in the inference of the GRN. The more omics layers not involved in the GRN inference, the more ambiguity exists in the network. This ambiguity leads to the unreliability and misinterpretation of the results obtained from the identified GRN.

The classic central dogma of molecular biology, i.e., “DNA makes RNA makes protein”, describes how genetic information travels from DNA to protein. In this paper, we consider the following three different omics layers in this traveling.

**Fig 2:**
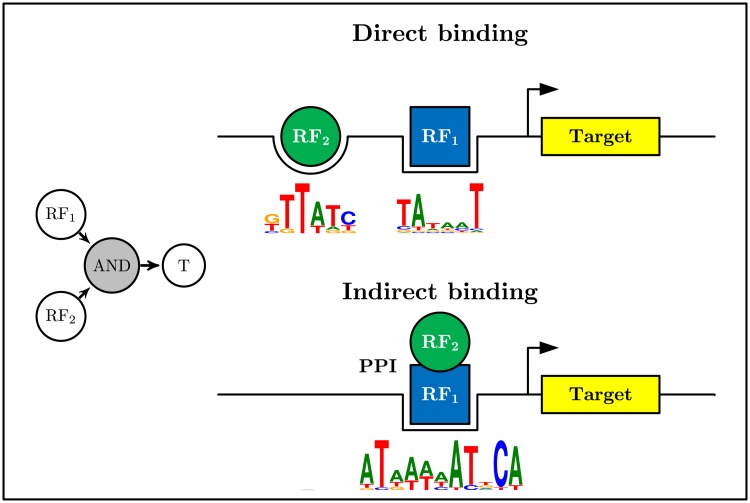
Direct vs. indirect RF-DNA binding. Both RF_1_ and RF_2_ are required for expression of the target gene T (AND logic). The RF-DNA binding resulting in the underlying AND logic can occur in two ways. A: In direct binding, both RFs have a binding site in the target promoter region. B: In indirect binding, one RF sits in its binding site, and the other RF influences T by a PPI with the directly bound RF. The sequence logos are sketched by WebLogo [25].

**Genome layer:** This layer is the lowest layer that can provide information about the GRN. The fundamental controlling elements in this layer are the *cis*-regulatory elements that are regions of non-coding DNA in the upstream of the transcription start site. The *cis*-elements control the spatial and temporal expression of nearby genes by functioning as the binding site of the active TFs (TFBS) and the associated co-factors. Most of the GRN architecture, referring to the topology of the functional linkages between the genes encoding TFs and their target genes, is directly specified by the *cis*-regulatory elements within the network [20]. In other words, a TF is considered as the regulator of a target gene if its related *cis*-element is next to that gene.
**Transcriptome layer:** By transcription of the genes and production of the mRNAs, we can move to the upper layer, i.e., the transcriptome layer. Transcription data, provided by different types of microarray technology or RNA-seq, can be used to monitor the expression level of many genes simultaneously and discover if the potential target genes are up- or down-regulated after each perturbation. The microRNAs (miRNAs) can be studied in this layer as well.
**Proteome layer:** After translation of the mRNAs to proteins, the protein interactions play their role in gene regulation. The RF proteins can interact with DNA either directly or indirectly [21]. Direct interaction involves binding an RF in the target promoter region, i.e., the *cis*-element discussed above. However, some RFs do not have a binding site and associate with DNA indirectly through protein-protein interaction (PPI) with already bound RFs (Fig 2). For instance, PPIs have a central role in strengthening a low-affinity TF-DNA binding and forming a high affinity. As an example, the E2F family TFs cannot bind DNA with high affinity but show high affinity to DNA by having PPI with DP family TFs and making E2F-DP heterodimer [22]. On the other hand, the retinoblastoma protein (pRb) can bind and inhibit the E2F-DP dimer [23]. Hence, *cis*-elements cannot reveal the regulatory effect of the indirectly bound RFs on the target genes because these RFs do not have a DNA biding site. In this case, PPI data can give insights regarding the network by revealing the regulations caused by indirectly bound RFs. For example, out of the 948 yeast RF_1_→T←RF_2_ regulatory interactions found as an AND logic gate, 348 have one indirect binding. Similarly, from 888 AND logic gates identified in the human leukemia dataset, 71 regulatory interactions have one indirect RF [24]. However, a PPI does not necessarily imply a regulatory interaction. In other words, a protein having a physical interaction with a bound RF can implicitly and optimistically be considered as a putative RF; otherwise, we cannot confirm its regulatory effect on the target gene by merely a physical interaction.
  In GRN inference, some spurious regulatory interactions that are indeed the indirect effect of the ancestors of a node, say a target gene, in the graph, are usually identified. Distinguishing these indirect effects from the direct effects of the parent nodes, say the regulatory genes, in the graph, is severely a challenging task in GRN inference, especially when we use only the transcriptomic data. In this case, the PPI between a previously proven parent RF and another RF could provide an evidence for the direct effect of the latter.

### Integrative perturbation analysis

In this paper, we analyze the logic-based Boolean modeling of the GRNs in two parts. In the first part, we analyze the effect of using different types of perturbations, i.e., knock-down and over-expression techniques, as well as of integrating different omics layers, in order to discover the missing regulatory interactions in the network and to deal with the visibility problem (by performing visibility analysis).

In the second part of our analysis, the ambiguity analysis will be performed. If we have a set of data to be utilized for the GRN inference, different models and graphs can be matched with the available data; however, only one of them will match with the reality. Hence, we use the term *ambiguity* here meaning that the real GRN model obtained by using a specific data set exists among many other unreal models. In other words, the more ambiguity exists in the results of the network inference, the more unreal network models are candidates to represent themselves as the real model. As was mentioned earlier, this ambiguity can be reduced by employing (i) different types of perturbations, i.e., knock-down and over-expression; (ii) multi-perturbation experiments versus single perturbations; and (iii) integration of multi-omics data. In the second part of our analysis, the effect of all of the above factors on the ambiguity will be investigated in our ambiguity analysis. First of all, we will introduce a step-by-step perturbation experiment that is equivalent to the multi-perturbation experiment. Then, we will analyze the effect of the single- and multi-step perturbation experiments for different types of perturbations as well as the data integration of different omics layers on the existing ambiguity in the logic-based GRN models.

The outcome of our analysis is an optimistic upper bound on some probabilities indicating the best possible performance. For example, provided that TF_1_ is the regulator of a target gene T, any physical PPI with TF_1_ does not necessarily mean that the interacting protein is also the regulator of T. However, as was explained previously, we optimistically consider it as an indirectly bound regulator of the target gene. In this optimistic analysis, we assume that we have a full knowledge of the omics layers, and, consequently, that the PPI and *cis*-element databases are complete without any missing data. We perform this optimistic analysis to show what level of certainty can be expected in the most optimistic scenario by using a GRN inference method employing different types of omics layers and perturbations. However, the performance of different inference methods in non-optimistic real cases would be equal or less than the upper bounds obtained in this paper.

Ultimately, by performing the above analysis, we try to shed light on the answers of the following questions:

1. What is the effect of the multi-perturbation experiments as well as the multi-omics data integration on the identification of the underlying Boolean functions?
2. How much ambiguity is eliminated from the Boolean GRN model by considering different perturbation types, more perturbations and more omics layers?
3. How accurate is the GRN modeled by a specific set of omics layers?
4. Which omics layers play the most important role in unambiguous modeling of the GRN?

## Methods

### Selection of Boolean functions

Provided that a combination of *k* regulatory factors regulates a target gene, their interactions can be expressed as a Boolean function representing the underlying logic of the corresponding node in the GRN. As was mentioned previously, each Boolean function can be represented by a truth table showing the output of the function for different combinations of the input states, i.e., the steady states of the regulators. One of the steps in inferring a logic-based GRN is identifying the Boolean function of each node. The perturbation of the arguments of the function, i.e., the regulators, can be utilized to identify the Boolean function. To identify the underlying Boolean function *f* uniquely, we need to observe the steady state of the target gene, called the output, for all different combinations of the values taken by the input arguments of *f*, called the inputs. 2^*k*^ different input combinations are sorted in the truth table of *f*.

In our analysis, we obtain different probabilities by using an exhaustive search over all Boolean functions. As was mentioned previously, there are $2^{2^{k}}$ Boolean functions with *k* inputs. Each of them can be denoted by $F_{b}^{k}$, where the Boolean function index *b* is the decimal representation of its output set (Table 1); i.e., $0\leq b\leq 2^{2^{k}}-1$. However, all Boolean functions are not included in our search space. In other words, for a specific value of *k*, some functions belong to the function set of the lower values of *k*, i.e., mappings in which one or more of the inputs are ineffective. For instance, $F_{60}^{3}$ is equivalent to ${\text{F}}_{6}^{2}$ because the third input is ineffective in the former. Table 1 shows the truth table of all 16 Boolean functions *f*(A,B) with *k* = 2, where A and B are the input Boolean variables. Out of the 16 available Boolean functions, 6 have only zero or one input variable, i.e., $F_{b}^{2}$ functions with *b* ∈ {0, 3, 5, 10, 12, 15}, representing the Boolean functions 0, A, B, $\bar{B}$, $\bar{A}$, and 1, respectively. Hence, theses functions are excluded in the analysis whose *k* equals 2. In this way, 10, 218, and 64594 distinct Boolean functions are included in the search space of *k* = 2, 3, and 4, respectively.

**Table 1:** The truth table of all Boolean functions with two input variables.

A	B	$F_{0}^{2}$	$F_{1}^{2}$	$F_{2}^{2}$	$F_{3}^{2}$	$F_{4}^{2}$	$F_{5}^{2}$	$F_{6}^{2}$	$F_{7}^{2}$	$F_{8}^{2}$	$F_{9}^{2}$	$F_{10}^{2}$	$F_{11}^{2}$	$F_{12}^{2}$	$F_{13}^{2}$	$F_{14}^{2}$	$F_{15}^{2}$
**0**	**0**	0	0	0	0	0	0	0	0	1	1	1	1	1	1	1	1
**0**	**1**	0	0	0	0	1	1	1	1	0	0	0	0	1	1	1	1
**1**	**0**	0	0	1	1	0	0	1	1	0	0	1	1	0	0	1	1
**1**	**1**	0	1	0	1	0	1	0	1	0	1	0	1	0	1	0	1
***f*** **(A,B)**		**0**	AB	$\text{A}\bar{B}$	**A**	$\bar{A}\text{B}$	**B**	A⊕B	A+B	$\bar{A}\bar{B}$	A⊙B	$\bar{B}$	$\text{A}+\bar{B}$	$\bar{A}$	$\bar{A}+\text{B}$	$\bar{\text{AB}}$	**1**

The truth table of all 16 Boolean functions *f*(A,B) having 2 input variables A and B are shown in the above table. In the $F_{b}^{2}$ notation of the Boolean functions, *b* is the decimal representation of the binary values in each column. The corresponding function of each column is displayed in the last row. Here, ⊕ and ⊙ denote the exclusive OR (XOR) and the exclusive NOR (XNOR) operations, respectively. In the last row, the excluded functions in the search space whose number of inputs is less than 2 are in boldface. Accordingly, 10 out of the 16 Boolean functions remain in the search space.

In typical Boolean networks, all possible Boolean functions are considered in the network. However, some studies define a set of rules to select the Boolean functions that are biologically meaningful [26, 27], i.e., that are likely to exist in the real world. The analysis of this paper can also be performed by using this more restricted set of Boolean functions. Therefore, the use of the more restricted set of functions results in less ambiguity than that resulting from the use of all Boolean functions.

### Single perturbation state diagram

**Fig 3:**
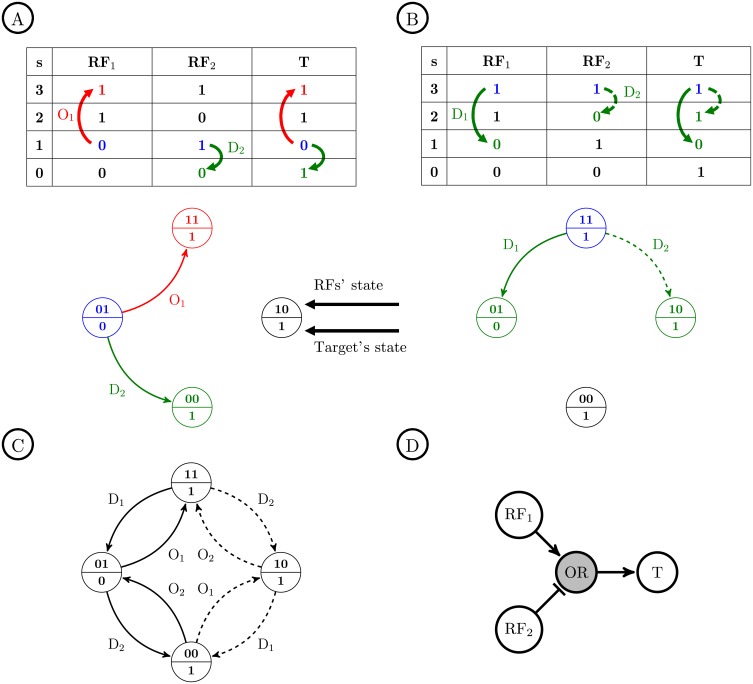
Single perturbation state diagram (SPSD) with two input variables. We introduce the SPSD to be able to show in a graph all state transitions of the regulators (RF_1_ and RF_2_) and the target gene (T) due to the knock-down (D) and over-expression (O) perturbations. The Boolean function $\text{T}\;=\;{\text{RF}}_{1}+\bar{\text{RF}2}$ is studied here. The blue, red, and green items correspond to the initial steady state, over-expression, and knock-down, respectively. A: The initial state of the inputs is 01. Both O_1_ and D_2_ perturbations cause a change in the state of T. Therefore, both corresponding edges in the SPSD are solid-line arrows. The upper and lower half of each node in the SPSD shows the state of the inputs and output, respectively. B: The initial state of the inputs is 11. D_2_ does not make any change in the output state; hence, the effect of RF_2_ on T is not visible (visibility problem), and the corresponding edge in the SPSD is a dashed-line arrow. C: The complete SPSD of the Boolean function for all possible initial states. D: The graph representation of the function $\text{T}\;=\;{\text{RF}}_{1}+\bar{\text{RF}2}$.

As was mentioned earlier, the goal of this paper is to analyze perturbation experiments in order to infer the GRNs. Hence, we introduce a state diagram, called the single perturbation state diagram (SPSD), representing all state transitions of a specific Boolean function for different perturbations, i.e., the knock-down and the over-expression, denoted by D and O, respectively. The SPSD of a Boolean function with *k* input variables consists of 2^*k*^ states (circle nodes) and the transition edges between them (see Fig 3). We note the binary sequence *b*_1_*b*_2_…*b*_*k*_ as the representation of the binary values of RF_1_, RF_2_, …, and RF_*k*_. The decimal equivalent of this binary sequence is called the *state number*, denoted by *s*. For instance, for a two-variable Boolean function, the state number of 2 represents the binary sequence 10, meaning that the state of RF_1_ and RF_2_ is 1 and 0, respectively. Now, we introduce the properties of the SPSD as follows.

**P1:** The upper half of each state node shows the state number (or the binary sequence) of the inputs and the lower half is the state of the output.
**P2:** Each edge is labeled by either D_*i*_ or O_*i*_, 1≤*i*≤*k*, denoting the knock-down or over-expression on the *i*-th RF. In other words, each state transition occurs by only a single perturbation.
**P3:** Each edge labeled by D_*i*_ (O_*i*_) makes a transition from a higher (lower) to a lower (higher) state number *s*.
**P4:** Provided that the single perturbation of an RF changes the state of the target gene, i.e., provided that we do not have the visibility problem, the corresponding transition edge in the SPSD will be displayed as a solid-line arrow; otherwise, it will be a dashed-line arrow.

Consider a two-variable Boolean function $\text{T}\;=\;{\text{RF}}_{1}+\bar{\text{RF}2}$ depicted in Fig 3. Assume that the initial state number is 1; i.e., the input states are 01. Accordingly, we can perform an over-expression on RF_1_ (O_1_) and a knock-down on RF_2_ (D_2_) to change their states and observe their effect on the target gene. As the truth table in Fig 3A reveals, by O_1_ and D_2_ we have the state transitions of 1→3 and 1→0, respectively. Moreover, the output state changes from 0 to 1 for both perturbations; hence, the effect of RF_1_ and RF_2_ on the target gene appears for both single perturbations. Therefore, for the above Boolean function, we do not have to deal with the visibility problem for *s* = 1. Some part of the SPSD corresponding to the transitions of *s* = 1 is shown in Fig 3A. According to the property P4, both edges coming out of this initial state are solid-line arrows.

Now assume that for the above Boolean function, the initial state number is 3; i.e., the initial states are 11 (see Fig 3B). In this case, we can knock down both RFs (D_1_ and D_2_) and observe 3→1 and 3→2 state transitions. For the latter, we have to deal with the visibility problem since the state of the output does not change with the D_2_ perturbation. Hence, the corresponding transition edge in Fig 3B is a dashed-line arrow. The complete SPSD showing all transitions for all initial states is displayed in Fig 3C, which reveals that the visibility problem is severely challenging for *s* = 2 because the effect of both RFs is invisible in this case.

**Fig 4:**
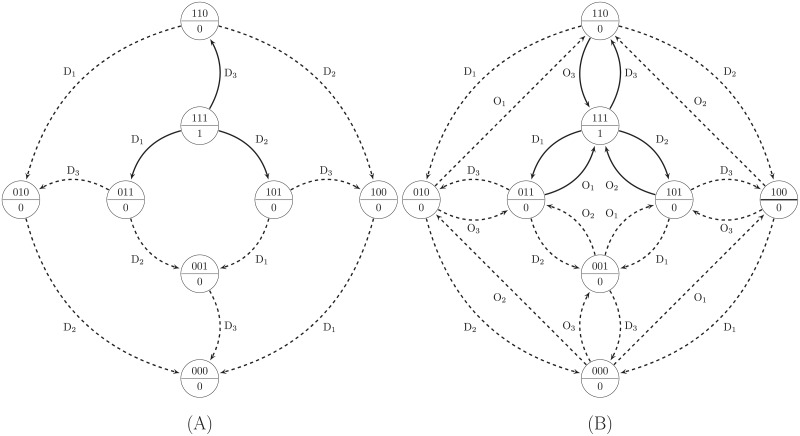
SPSD for the cases of the D and the DO perturbations. The SPSD of the 3-variable Boolean function T = RF_1_•RF_2_•RF_3_ is depicted for two types of perturbations experiments. A: Only knock-down is employed as the perturbation. Only edges labeled by D_*i*_ exist in the SPSD. B: Both knock-down and over-expression perturbations are used.

The SPSD introduced here will be used in the next sections to analyze the logic-based GRN inference. Moreover, two examples of the SPSD for *k* = 3 and 4 are depicted in Fig 4 and S1 Fig, respectively.

#### Finding the structure of the SPSD

The topology of the SPSD for all Boolean functions is the same for each *k*; i.e., the adjacency relation between the state nodes is fixed and is determined by the transitions between the states due to the perturbations. In this paper, two main categories for the perturbation experiments are considered:

1. Only the knock-down experiment (denoted by D);
2. Combination of the knock-down and over-expression experiments (denoted by DO).

The type of the perturbation experiment, i.e., either D or DO, affects the topology of the SPSD. In other words, in the case of using only the D perturbation, only transition edges labeled by D_*i*_, where 1≤*i*≤*k*, exist in the SPSD; otherwise, we will have edges labeled by both D_*i*_ and O_*i*_ in the SPSD (Fig 4). On the other hand, the visibility of the transition edges, i.e., the solid-line or dashed-line arrows, differentiates SPSDs from each other.

To analyze the effect of the data integration, the performance of the D and DO perturbation methods will be analyzed in three different following cases according to the type of the integrated data.

1. Only transcriptomic data (D and DO);
2. integration of transcriptome and PPI data (denoted by +PPI);
3. integration of transcriptomic data with either *cis*-element data, for direct RF binding, or a combination of *cis*-element and PPI data, for indirectly bound RFs (denoted by +cis(+PPI)).

Depending on the type of the integrated data, we have the following cases in regard to finding the structure of the SPSD.

1. In the case of using only the transcriptomic data (D or DO), the type of each transition arrow in the SPSD can be determined by using the truth table of the corresponding Boolean function. Strictly speaking, if the state of the output changes by transition to a new state node in the SPSD, the corresponding edge is considered as a solid-line arrow; otherwise, it will be a dashed-line arrow.
2. In the case of integrating the transcriptomic and PPI data (+PPI), if the effect of an RF on the target gene is invisible, and concurrently, this invisible RF shows a PPI with one of the visible RFs of the target gene, this invisible RF can be optimistically considered as an indirectly bound RF, and its transition edge can be converted to a solid-line arrow. In the SPSD, a dashed-line transition edge related to the perturbation of RF_*i*_ can be made visible if we have a pair of 1s in the state of either the source or the destination node of the transition edge, corresponding to RF_*i*_ and another visible RF. The pair of 1s is required because in a PPI, there are two interacting partners, and both of them should exist. In this case, we can optimistically assume that an interaction between these RFs will be found in the perfectly known PPI database revealing the effect of RF_*i*_ on the target gene. See the section Visibility analysis for more details.
3. If we also integrate both *cis*-element and PPI data for directly and indirectly bound RFs, (+cis(+PPI)), all transition edges are considered solid-line because all of them are optimistically visible in this case.

In the case of the DO experiments, for every edge labeled by D_*i*_, there is an edge labeled by O_*i*_ in the reverse direction. Hence, the solid and dashed edges in the SPSD are always symmetrical, except for the case of +PPI.

### Single- and multi-step perturbation experiments

In this section, we explain the details of the single- and multi-step perturbation experiments and their linkage with the SPSD. By perturbation of the input states, the genes interact until the network reaches a final cyclic attractor or a steady state. In this paper, we investigate only the case of reaching a steady state in the network; however, we will discuss briefly the generalization to cyclic cases at the end of this section. Moreover, we assume that the network is self-contained; i.e., any change in the network is due to the changes of other elements in the network. Therefore, in the process of identifying the GRN, every change in the states occurs because of the perturbations in our experiment.

**Fig 5:**
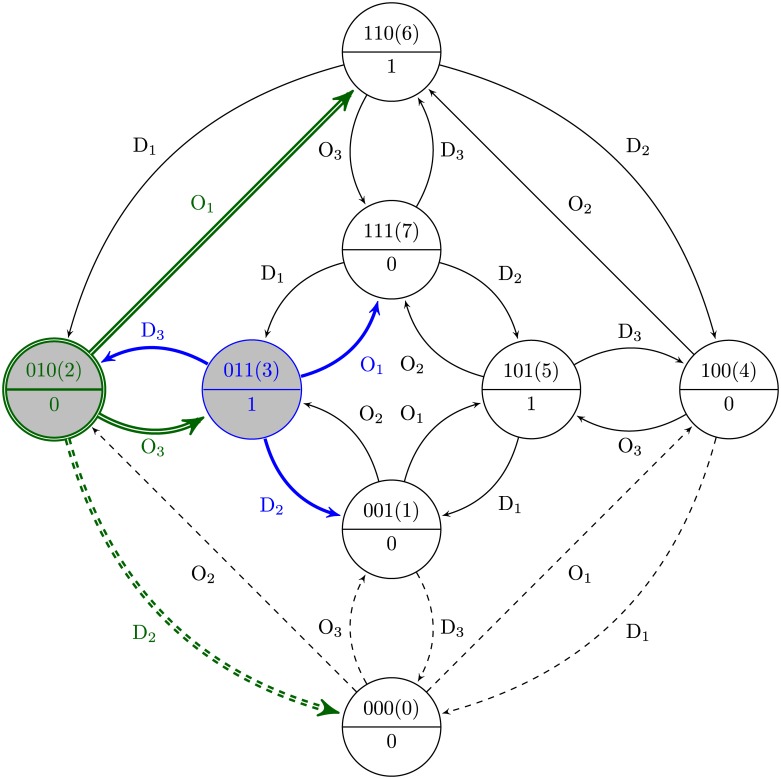
Multi-step perturbation experiment. The SPSD of the Boolean function $F_{22}^{3}$ is depicted. The single-line blue and double-line green nodes and edges are related to the first and the second step of the perturbation, respectively. Starting from the initial state number 3, we can perform the O_1_, D_2_, and D_3_ perturbations and switch to the state numbers 7, 1, and 2, respectively. Assume that we start the second step of the perturbation from the state number 2 and perturb by O_1_, D_2_, and O_3_. The two-step perturbation experiment performs as the multi-perturbation experiment perturbing two regulators simultaneously starting from the first initial state; i.e., the simultaneous perturbation pairs (D_3_,O_1_), (D_3_,D_2_), and (D_3_,O_3_) starting from *s* = 3 are equivalent to the two-step perturbation experiment starting from the *s* = 3 and 2, as the first and the second initial states, respectively.

The SPSD of the Boolean function $F_{22}^{3}$ is depicted in Fig 5. Assume that the initial state number is 3; i.e, the state of the inputs is 011. According to this initial state, we can perform the O_1_, D_2_, and D_3_ perturbations and switch from the state number 3 to the state numbers 7, 1, and 2, respectively (the thick single-line blue transition edges in Fig 5). Then, we can start the second step from any of the three state numbers 1, 2, or 7. Assume that we choose the state number 2 as the initial state of the second step. Accordingly, we display this state and the transition edges coming out of it by using the double-line green circle and arrows in Fig 5, respectively, representing the second step of the multi-step perturbation. In the second step, we should employ all O_1_, D_2_, and O_3_ perturbations; however, performing O_3_ is not required because it is the reverse of the transition 3→2 performed in the first step. Moreover, although the transition 2→0 is invisible in Fig 5, it can be considered visible because the effect of RF_2_ on the target gene was visible in the first step. In other words, the visibility is important only in the first step to detect the effective RFs on the target gene, and afterwards, we continue the next steps based on the identified RFs in the first step.

Further steps of the perturbation can be taken similarly from the states to which we switched. Note that in each step, we perform all *k* perturbations on the RFs and consider all corresponding transitions. For designing an experiment in practice, however, only one of the new states to which we transferred is chosen as the initial state of the next step. The new state that is intended to be the initial state of the next step may be biologically unstable. In this case, to observe the outcome of the perturbations of the next step, we can employ simultaneous multi-perturbations starting from the previous stable initial state. For example, in Fig 5, the goal of the second step of the perturbations starting from the state number 2 is observing the output of the state numbers 0, 3, and 6 (indeed, perturbing to observe the state number 3 is not required because it is already observed). Provided that the state number 2 is unstable, we can observe the output of the state numbers 0 and 6 by the simultaneous perturbation pairs (D_3_,D_2_) and (D_3_,O_1_), respectively, starting from the state number 3.

Provided that we deal with cyclic attractors rather than steady states, we can observe different combinations of the input states and the corresponding output state through the time. Hence, different states of the SPSD are observable over time without any perturbation (in the cases that the cycle exists inherently) or by triggering a cycle by a perturbation. Then, we can use this SPSD for identifying the Boolean functions. Therefore, in the cyclic cases, if we collect data over time as a time series, it can provide the same amount of information obtained by multi-perturbation experiments in the case of reaching steady states. However, there is a possibility that all 2^*k*^ input states do not occur in the cyclic attractors and we cannot observe those missing states in the time series.

### Analysis

In our analysis, multi-input motifs [28] with 2, 3, and 4 regulators are considered; i.e., the number of the inputs of the Boolean function $f_{i}\left(v_{i_{1}},...,v_{i_{k}}\right)$ of the *i*-th node or, equivalently, the in-degree of this node is 2≤*k*≤4. We analyze only the identification of the underlying Boolean function of a node in the GRN graph, as the main component of the graph, mapping the states of the inputs to the state of the single output. Accordingly, the results of this single-node analysis can be employed for analyzing the multi-node GRN graphs based on their specific architecture.

#### Obtaining the probability of visibility

The first question in our analysis is how employing different types of perturbation experiments and integrating different omics layers can diminish the visibility problem. In other words, we want to investigate how the above factors can provide more evidence for the existence of the invisible regulatory interactions and how much they increase the visibility. First of all, we want to learn, on average, what percentage of the transition edges of the SPSD over all Boolean functions and all initial states of their inputs is visible; i.e., we want to learn the probability of visibility.

As was mentioned previously, 10, 218, and 64594 Boolean functions are included in the search space of *k* = 2, 3, and 4, respectively, denoted by $N_{B}^{{}^{\left(k\right)}}$. Assume that $S_{B}^{{}^{\left(k\right)}}$ is a set of Boolean function indexes consisting of the *b* index of $N_{B}^{{}^{\left(k\right)}}$ functions included in the search space. To obtain the probability of visibility, *P_v_*, over all Boolean functions and all states of their SPSD, we use the following formula:

$$\begin{array}{c} P_{v}=\frac{1}{N_{B}^{{}^{\left(k\right)}}}\sum_{b\in S_{B}^{\left(k\right)}}(\frac{1}{k^{\prime }2^{k}}\sum_{s=0}^{2^{k}-1}N_{k,b,s}), \end{array}$$ (1)

where *N*_*k*,*b*,*s*_ is the number of the solid-line arrows coming out of the *s*-th state of the SPSD of $F_{b}^{k}$ and *k*′ equals *k*/2 and *k* for D and DO perturbations, respectively. The term *k*′2^*k*^ represents the total number of the transition edges in the SPSD, where, in the case of D, it is half of that in the case of DO.

Now, let us analyze the visibility in terms of the certainty in the GRN inference. The first condition for the certain determination of the underlying Boolean function of a node in the graph is the visibility of the regulatory interaction of all of its regulators. We call this property the *full visibility*. Equivalently, in the SPSD of the Boolean function, all transition edges coming out of the current state should be a solid-line arrow, meaning that they all should be visible. However, the visibility depends on the state number (or the steady state of the inputs) in the SPSD. Therefore, solid-line edges might be present for all perturbations in some state numbers while in others they may not. Hence, the outcome of the full visibility analysis here shows what percentage of the states in the SPSD, on average, over all states and all Boolean functions with *k* inputs have *k* solid-line transition edges coming out of them, i.e., that do not deal with the visibility problem at all. The percentage of fully visible nodes with the state number *s* over all Boolean functions can be calculated by

$$\begin{array}{c} P_{fv,s}=\frac{1}{N_{B}^{{}^{\left(k\right)}}}\sum_{b\in S_{B}^{\left(k\right)}}I\left(N_{k,b,s}=k\right), \end{array}$$ (2)

where I(*N*_*k*,*b*,*s*_ = *k*) is an indicator function taking the value of 1 if *N*_*k*,*b*,*s*_ equals *k*; otherwise, it will be zero. Then, the probability of full visibility, *P_fv_*, which is averaging over all Boolean functions and all states can be calculated by the following formula:

$$\begin{array}{c} P_{fv}=\frac{1}{2^{k}}\sum_{s=0}^{2^{k}-1}P_{fv,s}. \end{array}$$ (3)

#### Obtaining the probability of unambiguity

As was mentioned previously, one of the sources of ambiguity in GRN inference is that several models matching with our specific data set are candidates for representing themselves as the real model, but only one of them is the real one, and the others are unreal. In this ambiguity analysis, we obtain the number of the Boolean functions that match with the data set for different types of the perturbation and the integrated data. Then, we find the probability of unambiguity, denoted by *P_ua_*, which is the probability of getting the real model out of the candidates.

In the SPSD of the Boolean function ${\text{F}}_{b}^{k}$, for each initial state, we can consider a set consisting of the output of the source and destination states of all fully visible transitions coming out of the initial state. This set is common in the SPSD of several Boolean functions. The number of these functions having a common set of transitions is denoted by $N_{k,b,s}^{{}^{\text{cmn}}}$. However, only one of these functions is the real representative of the underlying regulatory interaction. Hence, the probability of choosing the real Boolean function from among these functions is $1/N_{k,b,s}^{{}^{\text{cmn}}}$. Accordingly, the probability of unambiguity, *P_ua_*, over all Boolean functions and all different initial states of their inputs can be obtained as follows:

$$\begin{array}{c} P_{ua}=\frac{1}{2^{k}N_{B}^{{}^{\left(k\right)}}}\sum_{b\in S_{B}^{\left(k\right)}}\sum_{s=0}^{2^{k}-1}\;\;\frac{I(N_{k,b,s}=k)}{N_{k,b,s}^{{}^{\text{cmn}}}}, \end{array}$$ (4)

where I(*N*_*k*,*b*,*s*_ = *k*) in the above equation guarantees that for each SPSD, we consider only the initial states whose transition edges coming out of them are all visible.

As was mentioned earlier, we are performing an optimistic analysis in this paper. In the case of multi-step perturbation, we consider all possible paths in the SPSD and then choose the ones with the least ambiguity. For instance, consider the example depicted in Fig 5, which has three different paths of 3→1, 3→2, and 3→7 as the first step. Then, the second step has three more paths for each of the above paths. For example, by considering the state number 2 as the second initial state number, we have paths 3→2→0, 3→2→3, and 3→2→6 for the ordered pair of initial state numbers (3,2). The corresponding paths of the ordered pairs of initial state numbers (3,1) and (3,7) can be found similarly. Ultimately, to find the best candidate in our optimistic analysis, we consider the ordered pair of initial state numbers and their corresponding transitions whose ambiguity or, equivalently, $N_{k,b,s}^{{}^{\text{cmn}}}$, is minimum. Note that all transitions starting from the first step up to the last one are considered in determining $N_{k,b,s}^{{}^{\text{cmn}}}$.

## Results and discussion

### Visibility analysis

Provided that a transition edge in a SPSD is represented by a dashed-line arrow, we can consider it as a solid-line arrow if other omics layers can give evidence of the effect of its corresponding RF on the target gene. Indeed, data integration can reduce the visibility problem by increasing the number of the solid-line arrows in the SPSD. The more solid-line arrows observed in the SPSD, the less visibility problem exists. Accordingly, we use the following integrative rules as the evidence for the existence of a regulatory interaction. The regulatory effect of RF_*i*_ on a target gene is visible if

**R1:** a perturbation D_*i*_ or O_*i*_ on RF_*i*_ in the SPSD changes the state of the target gene;
**R2:** the corresponding *cis*-element of RF_*i*_ exists in the upstream of the target gene;
**R3:** RF_*i*_ has a physical PPI with a previously proven regulator of the target gene.

**Fig 6:**
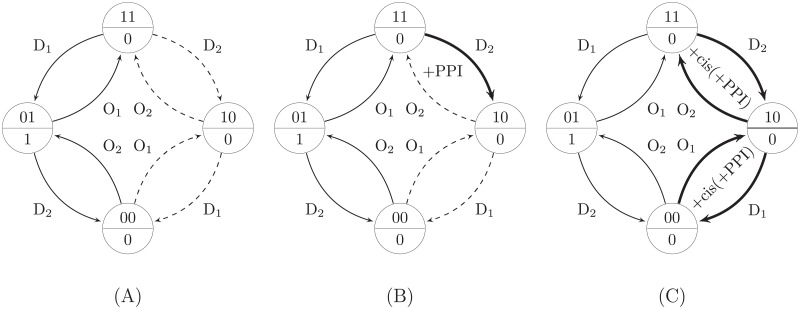
The effect of data integration on visibility. Data integration can reduce the visibility problem by making some invisible (dashed-line) edges in the SPSD visible (solid-line). The edges newly revealed by using data integration are highlighted by the thick edges. The SPSD of the Boolean function $f\left(\text{A},\text{B}\right)=\bar{\text{A}}\text{B}$ is shown for integrating different types of data. A: Only transcription data (rule R1). B: Transcription data+PPI (rules R1 and R3). C: Transcription data+cis(+PPI) (rules R2 and R3).

In Fig 6, we can see the effect of applying the above rules on the visibility. Fig 6A shows the SPSD of the Boolean function $f\left(\text{A},\text{B}\right)=\bar{\text{A}}\text{B}$ in the case of using only the transcriptomic data applying only R1. In this SPSD, 4 out of the 8 edges are invisible. Assume that the initial state is 11; according to Fig 6A, we have an evidence for the effect of RF_1_ on the target gene and the effect of RF_2_ is not visible in this state. However, if we have a PPI between RF_1_ and RF_2_, we can consider the D_2_ edge coming out of the state 11 as visible by applying R3 (the thick edge in Fig 6B). But for the initial state 10 we do not have any evidence for the effect of RF_1_ on the target gene; hence, any PPI between RF_1_ and RF_2_ cannot help to reveal more regulatory interactions for this initial state. Therefore, the O_2_ edge coming out of the state 10 remains invisible. On the other hand, Fig 6C shows that we can convert all invisible edges to visible by integrating the *cis*-element and PPI data (combination of rules R2 and R3 for directly and indirectly bound RFs).

**Fig 7:**
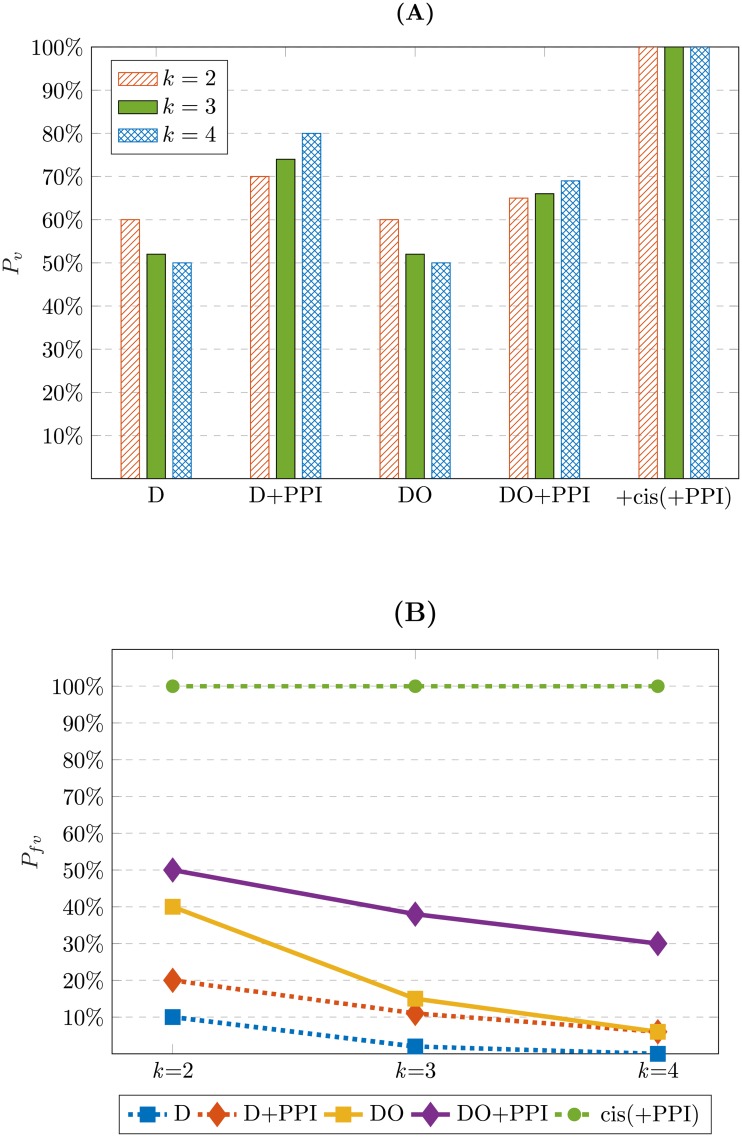
The results of the visibility analysis. The effect of employing different types of perturbations and integrating different omics data is depicted for *k* = 2, 3, and 4. The average percentages or probabilities are over all Boolean functions and all initial states. A: Probability of visibility (*P*_*v*_), i.e., the average percentage of the visible edges in the SPSD. B: Probability of full visibility (*P*_*fv*_), i.e., the average percentage of the nodes in the SPSD with full visibility; i.e., all edges coming out of them are visible (solid-line arrows).

The results of the visibility analysis obtained by applying the above rules are depicted in Fig 7. The probability of visibility of the transition edges in the SPSD, i.e., the average percentage of the visible edges over all Boolean functions and all initial states, is shown in Fig 7A for various combinations of the perturbation experiment and omics layer data as well as different numbers of the inputs of the Boolean functions (*k*). The values of the probabilities are shown in S1 Table. As Fig 7A shows, provided that we use only the transcriptomic data, i.e., the cases of D and DO, for each *k*, *P*_*v*_ is the same for both cases. The reason is that for every edge labeled D_*i*_, there is an edge in the reverse direction labeled O_*i*_; hence, as Fig 4 demonstrates, the number of the transition edges with D is half of that with DO. Therefore, by using only knock-down experiments, only the edges labeled D_*i*_ remain in the SPSD (Fig 4A). However, it was mentioned in the section Methods that the solid and dashed edges in the SPSD of DO are symmetric. Hence, although the number of the edges in the SPSD of D is half of that in the SPSD of DO, both SPSDs have the same portion of the visible edges, resulting in the same amount of *P*_*v*_.

As Fig 7A reveals, for both cases of D and DO, by combining more omics layers we can make a larger portion of the edges visible. As was explained earlier, most of the network architecture can be captured by using *cis*-elements. If there are indirectly bound RFs without a DNA binding site, we can optimistically expect to observe the invisible part of the architecture by utilizing the physical PPIs between the invisible RFs and the previously proven RFs identified by using *cis*-element data. Therefore, the optimistic probability of visibility for the case of +cis(+PPI) is 100% in Fig 7A. Strictly speaking, in the case of having a full knowledge of PPIs and *cis*-elements without any missing data, it does not matter if we employ either D or DO perturbations, and the combination of *cis*-elements and PPI data can optimistically tell the whole story about the network architecture. In other words, all transition edges in the SPSD become solid-line arrows in this case, so that all of the edges are visible. Here, +cis(+PPI) is considered as the best case scenario for the specific set of multi-omics data used in our analysis, i.e., *cis*-element, transcription, and PPI data, based on the assumption that all PPI and *cis*-element information is perfectly known and we have no failure in the transcriptomic data. Definitely, we need to use the data of more omics layers, e.g., epigenetic or phenotypic data, if we have failure or missing information in our databases. Therefore, the best case scenario differs for different combinations of the multi-omics data. In conclusion, it is recommended that in reality, researchers use at least the *cis*-element and PPI data in the first step of the GRN inference process to capture either all or most of the architecture of the network.

### Full visibility analysis

As was discussed in the section Methods, having the full visibility property is necessary for certain determination of the Boolean function of a node in the graph. In other words, all transition edges coming out of the current state of the SPSD should be solid-line arrows. Therefore, we perform the full visibility analysis here to show what percentage of the states in the SPSD, on average, over all Boolean functions with *k* inputs have the full visibility property; i.e., the probability of being fully visible for a state in the SPSD. The results are sketched in Fig 7B showing the probability of full visibility, *P*_*fv*_, for different values of *k* and various integrative perturbations. The values of the probabilities are visible in S1 Table.

Fig 7B shows that only the knock-down perturbation (D) has the lowest probability of full visibility. Indeed, the first condition to have full visibility by the D perturbation is that the steady state of all RFs should be 1 to be able to knock them down. In other words, in Eq (2), the term I(*N*_*k*,*b*,*s*_ = *k*) can be 1 only for the highest state number, i.e., *s* = 2^*k*^-1, and consequently, in Eq (3), only *P*_*fv*,*s* = 2^*k*^−1_ is nonzero. Hence, only one node in the SPSD can lead to full visibility, and all other states cannot result in a full recognition of all regulators. Consequently, the percentage of the nodes in the SPSD having full visibility over all Boolean functions and all initial states will decrease dramatically if only the knock-down perturbation is employed. Similarly, in the case of D+PPI, we are still limited to the highest state number in the SPSD as the required initial state for having full visibility and *P*_*fv*_ remains low, although it would be more than only D.

We can use the DO perturbation to eliminate the limitation of the D perturbation for the full visibility. In this case, the process of the GRN inference can be started from all 2^*k*^ initial states of the SPSD because the state of the regulators can be perturbed with any steady state of either 0 or 1. Due to the involvement of all 2^*k*^ states of the SPSD in DO, the probability of having full visibility increases significantly by a factor of 2^*k*^ compared to D. This probability can be increased even more by integrating the PPI data and using the rule R3, i.e., DO+PPI.

As was defined previously, *P*_*fv*_ shows the probability of being a fully visible node in the SPSD over all Boolean functions and all initial states. With the knowledge that in the cases of D and D+PPI, full visibility can occur only in the top state, i.e., *s* = 2^*k*^ − 1, one may ask what percentage of the top states over all Boolean functions are fully visible by using different data types. To answer this question, we should obtain the value of *P*_*fv*,*s* = 2^*k*^−1_ by using Eq (2). As was mentioned above, in the case of D and D+PPI, *P*_*fv*,*s*_ is non-zero only for *s* = 2^*k*^ − 1. Consequently, Eq (3) shows that in the case of D, the value of *P*_*fv*,*s* = 2^*k*^−1_ is 2^k^ times of the corresponding value of *P*_*fv*_. In addition, in the case of D+PPI, 80%, 89%, and 94% of the top states would be fully visible for *k* = 2, 3, and 4, respectively. The values of *P*_*fv*,*s*_ for different values of *k* and all state numbers are listed in S2 Table. In the case of DO, all states have the same amount of *P*_*fv*,*s*_. However, in the case of DO+PPI, the more number of 1s in the binary representation of *s* results in the larger value of *P*_*fv*,*s*_. Indeed, the more number of 1s means the more possibility for observing PPIs, resulting in the increase of the probability of full visibility for the state node. Moreover, the value of *P*_*fv*,*s* = 2^*k*^−1_ in the cases of D and D+PPI is the same as the corresponding value of *P*_*fv*,*s* = 2^*k*^−1_ in the cases of DO and DO+PPI.

As Fig 7B shows, although DO+PPI increases *P*_*fv*_ significantly compared to D, D+PPI, or DO, in the best case that *k* = 2, more than 50% of the nodes in the SPSD over all Boolean functions and all states are not still fully visible. This gap becomes even larger by increasing *k*. As was mentioned previously, we should use the integration of the *cis*-element and PPI data to capture the full architecture of the network, so that all states in the SPSD will be fully visible in the case of +cis(+PPI). In other words, in Eq (2), *N*_*k*,*b*,*s*_ equals *k* for all states, resulting in *P_fv,s_* = 1 for all values of *s*, and *P_fv_* = 1, regardless of the value of *k* and the type of D or DO. In other cases, Fig 7B shows that *P*_*fv*_ decreases remarkably with the growth of *k*.

In conclusion, according to the above discussion, the level of visibility in identifying the underlying Boolean functions of the logic-based model of the GRN is severely affected by the type of perturbation and data employed for the GRN inference. If we do not choose the type of the multi-omics data wisely and do not employ the required types of perturbations in our experiments, the results of the GRN modeling will suffer dramatically from missing edges leading to unreliability and spuriousness. Consequently, the predictions obtained by this incomplete GRN model will be inaccurate and misleading.

The results of this analysis can also be generalized to the inference of the interactive networks. The inference methods of these networks also encounter the visibility problem, and our results can lead to developing guidelines for them as well.

### Ambiguity analysis of integrative single perturbation

As we did in the previous analysis, we analyze the identification of the underlying Boolean function of a single node in the GRN graph with *k* inputs (2≤*k*≤4). In this part, we obtain the number of the Boolean functions that match with the data set for different types of the perturbation and the integrated data. Then, we find the probability of unambiguity, *P_ua_*, which is the probability of getting the real model out of the candidates. However, we are particularly interested in finding these probabilities, on average, over all Boolean functions and all different initial states of their inputs. More details of obtaining *P_ua_* were discussed in the Methods section.

**Fig 8:**
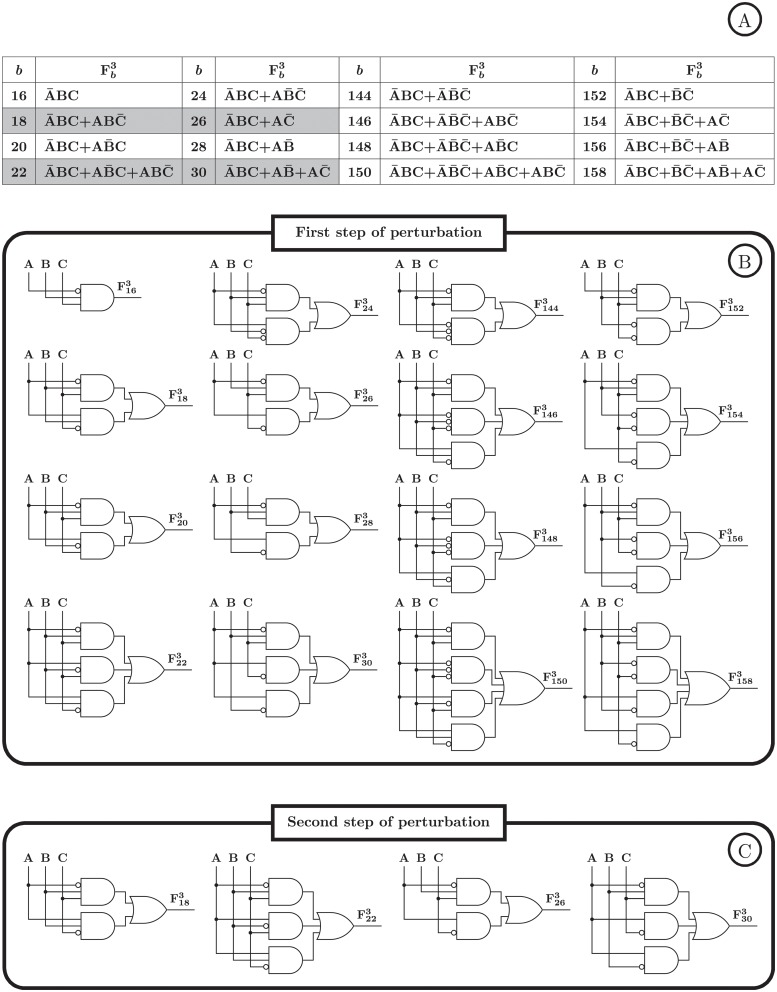
Ambiguity in logic-based GRN modeling. We consider the underlying Boolean function of a regulatory interaction as $F_{22}^{3}$; i.e., $f\left(\text{A},\text{B},\text{C}\right)=\bar{\text{A}}\text{B}C+\text{A}\bar{\text{B}}\text{C}+\text{AB}\bar{\text{C}}$. A: The set of the transitions in the SPSD of $F_{22}^{3}$ starting from the initial state number 3 (Fig 5) can be observed in the SPSD of 15 other Boolean functions. All these functions are listed in the table. By taking the second step of the perturbation from the state number 2, the number of the Boolean functions having the common set of transitions is reduced to 4. The gray cells in the table are the remaining functions after taking the second step. B: The logic circuit of the functions listed in the table. C: The logic circuit of the remaining functions after the second step (corresponding to the gray cells in the table).

First, the integration of the single perturbation with multi-omics data will be analyzed here. A set of transitions can be observed by starting from the initial state in the SPSD of the real Boolean function. Similarly, this specific set of transitions can also be observed in the SPSD of some other Boolean functions for the same initial state. For example, the set of transitions of the SPSD of $F_{22}^{3}$ (Fig 5) for *s* = 3 with the single DO perturbation is common in the SPSD of 16 Boolean functions $F_{b}^{3}$ where *b* ∈ {16, 18, 20, 22, 24, 26, 28, 30, 144, 146, 148, 150, 152, 154, 156, 158}; i.e., $N_{3,22,3}^{{}^{\text{cmn}}}=16$. The logic representation of the above Boolean functions and their corresponding logic circuits are displayed in Fig 8A and 8B, respectively.

**Fig 9:**
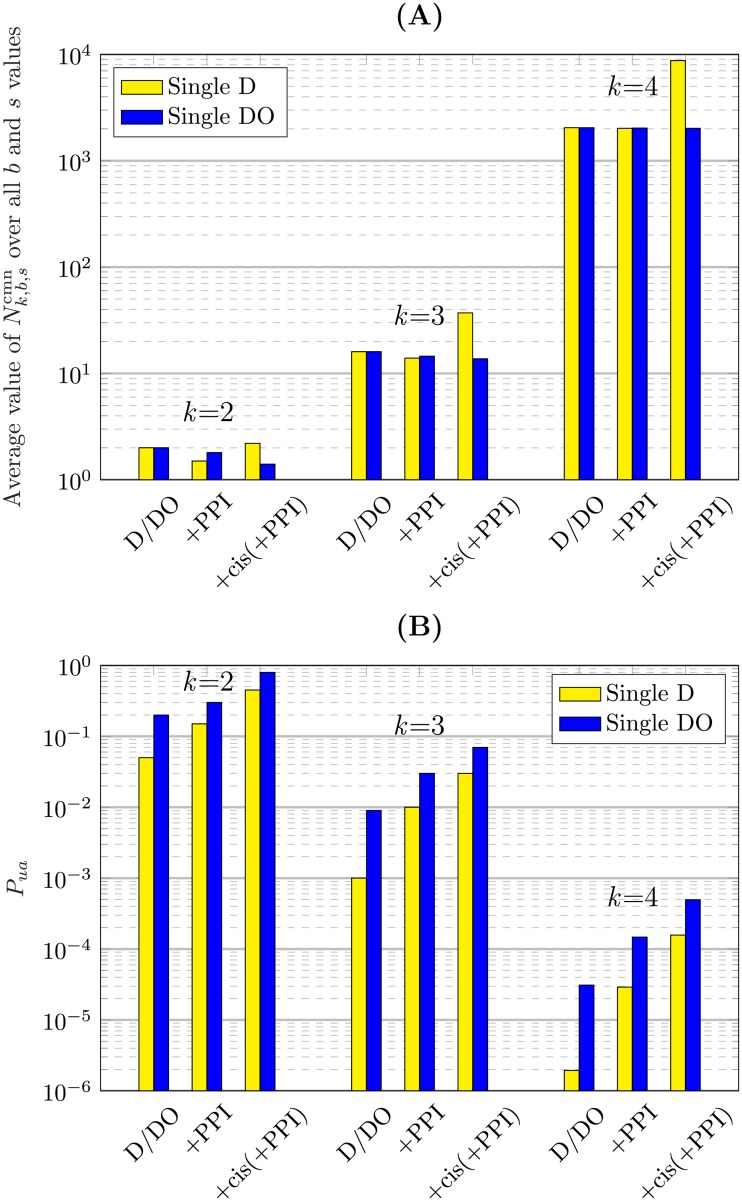
Ambiguity analysis of integrative single-step perturbation. The analysis is performed for the D and the DO perturbations in integration with different types of omics data over all Boolean functions and all initial states for *k* = 2, 3, and 4. A: One of the sources of the ambiguity in logic-based GRN inference is that the set of the transitions in the SPSD due to the perturbations is common between $N_{k,b,s}^{\text{cmn}}$ number of the Boolean functions, which only one of them is the real one. The average value of $N_{k,b,s}^{\text{cmn}}$ over all values of *b* and *s* is displayed for different values of *k*. B: The probability of unambiguity (*P_ua_*) for integrative single-step perturbation.

The average value of $N_{k,b,s}^{{}^{\text{cmn}}}$ over all values of *b* and *s* and for different values of *k* is displayed in Fig 9A (S3 Table presents the corresponding values of this figure). Note that for D and D+PPI only *s* = 2^*k*^-1 is considered. Obviously, the number of these Boolean functions grows dramatically with the growth of *k*. Consequently, an increase in *k* provides more unreal candidates for the underlying Boolean function that can match the data set, resulting in more ambiguity. This fact is also observable in Fig 9B which shows that *P_ua_* decreases significantly by *k*. The values of the probabilities depicted in Fig 9B are given in S4 Table.

Now, let us study the effect of employing different types of perturbations, i.e., D and DO, on the ambiguity. Fig 9B shows that in a comparison between D and DO, the latter results in a more unambiguous identification of the Boolean functions. Without any integration with other omics layers, *P_ua_* for DO is 4, 8, and 16 times more than that of D for *k* = 2, 3, and 4, respectively, i.e., 2^*k*^ times more. The reason is that, as was explained in the Full visibility analysis section, the full visibility which is necessary for certain identification of the Boolean function occurs in the case of D only for one out of the 2^*k*^ states of the SPSD, resulting in 2^*k*^ times more ambiguity compared to DO. In other words, in the case of the D perturbation in Eq (4), the second summation is effective only for *s* = 2^*k*^-1 making the probability of unambiguity 2^*k*^ time less than that of DO which all 2^*k*^ state numbers are effective in the summation.

Fig 9B also reveals the effect of the multi-omics data integration on ambiguity. As we expected, +cis(+PPI) has the most effect on eliminating the ambiguity and the impact of the integration of only PPI data is less. Since the vertical axis of Fig 9B is a logarithmic scale, and most of the curves in this figure are approximately linear, we can conclude that integration of different omics layers increases the probability of unambiguity exponentially. Therefore, including as many omics layers as possible in the GRN inference makes the results more accurate, unambiguous, and trustworthy. On the other hand, we should be more doubtful about the results of the studies employing either a single data type or a small number of the omics layers.

As Fig 9B shows, the highest value of *P_ua_* occurs for the case of DO+cis(+PPI) with *k* = 2, which is 80%. Although +cis(+PPI) significantly increases *P_ua_*, the identification of the Boolean functions is still not completely unambiguous and certain. To decrease ambiguity and uncertainty, we need to perform multi-perturbation experiments employing two or more perturbations. These experiments are analyzed in the next section.

### Ambiguity analysis of integrative multi-step perturbation

As was explained in the previous section, one of the sources of the ambiguity in logic-based GRN models is that the transitions observed in our experiment are common in the SPSD of several Boolean functions (Fig 8). To deal with this problem, the experiment should be designed to observe more transitions and states of the SPSD. Consequently, the observed transition set will be in common with fewer of the Boolean functions, resulting in less ambiguity. If the experiment covers all states and transitions of the SPSD, the identified Boolean function will be unique. For example, in the case of the single perturbation, the DO experiment observes more transitions and states of the SPSD than the D, and hence, the DO performs less ambiguously than the D. Similarly, by employing single perturbations in several consecutive steps, we can expand over the SPSD and reduce the ambiguity. Indeed, by this approach, we will have a multi-perturbation experiment which applies the step-by-step single perturbations. By taking more steps in multi-step perturbation, less Boolean functions have common set of transitions in their SPSDs; i.e., $N_{k,b,s}^{{}^{\text{cmn}}}$ reduces by increase of the number of the steps, denoted by *n*_*p*_, resulting in less ambiguity. For example, for $F_{22}^{3}$ mentioned before, by taking the first and second steps starting from *s* = 3 and 2, respectively, the set of all transitions in the first and the second steps is common between four functions $F_{b}^{3}$ with *b* ∈ {18, 22, 26, 30}, which is 4 times lower than the number of the functions in the case of single perturbation (Fig 8C). For more details about this multi-step perturbation procedure, see the Methods section.

**Fig 10:**
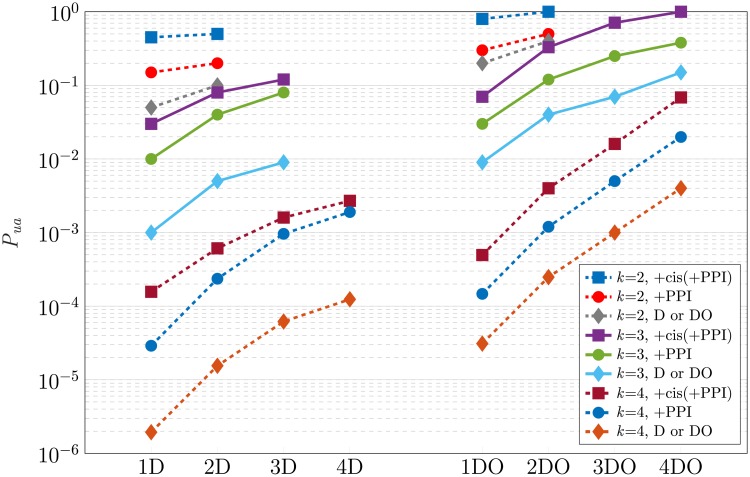
Ambiguity analysis of integrative multi-step perturbation. The probability of unambiguity is obtained for different number of the steps of the D and the DO perturbations (up to *n*_*p*_ = 4) in integration with different types of omics data over all Boolean functions and all initial states for *k* = 2, 3, and 4.

Fig 10 displays the results of the integrative multi-step perturbation analysis for two distinct cases of the perturbation, i.e., D and DO, depicted in two separate columns of curves. Multi-step perturbations up to four step-by-step single perturbations have been applied in each case in integration with different omics layers. The logarithmic scale of the probability of the unambiguity is depicted for *k* = 2, 3, and 4 against the number of the steps, *n*_*p*_. The notation *n*_*p*_D and *n*_*p*_DO, e.g., 2D and 4DO, denotes *n*_*p*_ steps of the D and the DO perturbation, respectively. The corresponding values displayed in Fig 10 are listed in S4 Table. The effect of different factors on the ambiguity of the GRN inference can be listed as follows.

#### Effect of *k*

In each of the D or DO cases, the ambiguity grows dramatically with the growth of *k*. By increasing *k* from 2 to 3 and 3 to 4 for different circumstances, *P_ua_* reduces in the order of 0.1-0.01 and 0.01-0.001 times, respectively.

#### Effect of data integration

The effect of the integration of the transcriptomic data with other omics data is observable in Fig 10. Employing only transcription data causes the most ambiguity for each *k*, and the integration with the *cis*-element data in combination with PPI data (+cis(+PPI)) results in the lowest ambiguity. Integration of the PPI and transcriptomic data performs better than the use of only the latter data; however, the performance is still less than that of +cis(+PPI). According to Fig 10 or S4 Table, the average unambiguity ratio, i.e., the ratio of the probabilities of integrative to non-integrative inference (i.e., using only transcriptomic data) over different values of *n*_*p*_ is shown in Table 2. For example, the value in the intersection of the D row and +PPI column means the average value of the ratios *P_ua_*(D+PPI)/*P_ua_*(D) over different numbers of the perturbations. The larger value in the table means that more ambiguity is eliminated by data integration. This table shows that data integration has a significant effect on reducing the ambiguity in the inferred GRN. This reduction in ambiguity becomes more effective by increasing *k*.

**Table 2:** The effect of data integration on the average unambiguity ratio of integrative to non-integrative inference over different numbers of perturbations.

	*k* = 2		*k* = 3		*k* = 4	
	+PPI	+cis(+PPI)	+PPI	+cis(+PPI)	+PPI	+cis(+PPI)
**D**	2.5	7	8.1	18	15.3	42
**DO**	1.4	3.3	3	8.4	4.9	16.5

The average ratios of *P_ua_*(D+PPI)/*P_ua_*(D) and *P_ua_*(D+cis(+PPI))/*P_ua_*(D) and the similar ratios for the DO perturbation are shown for different values of *k*. The average ratio is over different numbers of the perturbations; i.e, *n*_*p*_ equals 1 to 4, in the multi-step perturbation experiment. The larger value in the table shows that more ambiguity is eliminated by data integration.

#### Effect of different types of perturbations

In Fig 10, a pair of points with one belonging to the D perturbation and the other to the DO are called the *dual points* if they have been analyzed under the same circumstances other than the perturbation type. Overall, every point in Fig 10 corresponding to the DO experiment shows a higher probability of unambiguity than that of its dual point in the D perturbation. As was explained previously, in D experiments we can potentially have full visibility only if the initial state is the highest state number of the SPSD, resulting in more ambiguity compared to the DO. However, in the case of DO, we can reduce the ambiguity by observing more states and transitions in the SPSD.

Table 3 shows the ratio of the probability of the unambiguity of DO to that of D for the dual points and for different types of the integrated data and the multi-step perturbations; e.g., the columns labeled by DO/D and +PPI show the ratios Pr(DO)/Pr(D) and Pr(DO+PPI)/Pr(D+PPI), respectively. The values of *n*_*p*_ and *k* vary in the range of 1 to 4 and 2 to 4, respectively. The column DO/D in Table 3 shows that the ratio of the probabilities for the case of using only the transcriptomic data equals 2^*k*^ for 1≤*n*_*p*_≤3 and twice that for *n*_*p*_ = 4. The factor of 2^*k*^ is due to the involvement of all 2^*k*^ states of the SPSD having full visibility in the case of DO. This involvement increases the probability of having full visibility by a factor of 2^*k*^, resulting in more *P_ua_* compared to the case of D. Moreover, for *n*_*p*_ = 4, employing four steps of perturbation causes a reduction by a factor of 2 in the number of the Boolean functions having common transitions in their SPSDs. Therefore, in Table 3, the probabilities of DO/D for *n*_*p*_ = 4 are twice those for other values of *n*_*p*_.

**Table 3:** The effect of the multi-step perturbations on the unambiguity ratio of DO to D for different types of data integration.

	*k* = 2			*k* = 3			*k* = 4		
*n*_*p*_	DO/D	+PPI	+cis(+PPI)	DO/D	+PPI	+cis(+PPI)	DO/D	+PPI	+cis(+PPI)
**1**	4	2	1.8	8	3.3	2.4	16	5.1	3.2
**2**	4	2.5	2	8	3.2	4.3	16	5.1	6.5
**3**	-	-	-	8	3.1	5.7	16	5	10.3
**4**	-	-	-	16	4.9	8	32	10.5	25.4

The unambiguity ratios of *P_ua_*(DO)/*P_ua_*(D), *P_ua_*(DO+PPI)/*P_ua_*(D+PPI), and *P_ua_*(DO+cis(+PPI))/*P_ua_*(D+cis(+PPI)) are shown for different numbers of the steps of the perturbation (*n*_*p*_). These ratios reveal that employing the DO perturbation reduces the ambiguity significantly compared to the D perturbation.

The columns labeled by +PPI and +cis(+PPI) in Table 3 shows that utilizing the data integration of other omics layers with the results of the DO perturbation similarly leads to a higher probability of unambiguity compared to that of the D perturbation.

#### Effect of multi-step perturbation

As we also expected intuitively, Fig 10 shows that in all cases, the increase in the number of the perturbations, *n*_*p*_, reduces the ambiguity significantly. For multi-step D perturbations starting from the only fully visible initial state, i.e., *s* = 2^*k*^-1, after *n*_*p*_ = *k* perturbations we will reach the state number of 0 as the final destination. After that, we cannot go further because we would need an over-expression for the transition from that state. Consequently, for every kind of data integration with the results of the D perturbations, *n*_*p*_ = *k* provides the most information. Hence, for *k* = 2 and 3 in Fig 10, *P_ua_* does not exist for 3D and 4D, respectively.

In the case of the multi-step DO perturbations, for every *k*, if we continue increasing the number of the steps *n*_*p*_ from the initial states that are fully visible, all the states of the SPSD will be observed after a specific number of the perturbations. For instance, when *k* equals 2 and 3, the whole SPSD is observed, while *n*_*p*_ is equal to 2 and 4, respectively. In this case, no other Boolean function has a common set of transitions with the real function, i.e., $N_{k,b,s}^{{}^{\text{cmn}}}=1$; therefore, the Boolean function $F_{b}^{k}$ can be identified uniquely. While $N_{k,b,s}^{{}^{\text{cmn}}}=1$, *P_ua_* in Eq (4) is equivalent to *P_fv_* in Eq (3). Therefore, the most achievable *P_ua_* for multi-step DO perturbation is the corresponding probability of the full visibility. According to the results of the full visibility analysis displayed in Fig 7B, the maximum probability of the full visibility occurs for the case of DO+cis(+PPI), which is 100%. Consequently, for the the case of DO+cis(+PPI), *P_ua_* can reach 100% for *k* = 2 and 3 while *n*_*p*_ = 2 and 4, respectively. More perturbations are required for *k* = 4 to reach 100% probability. According to Fig 7B, the most achievable *P_ua_* for the cases of DO and DO+PPI will be less than 100%. We can reach 100% unambiguous identification of the Boolean function by using multi-step DO perturbation because unlike D, DO is not caught in the trap of the state number of 0; hence, the transitions can go further, causing the full observation of the SPSD for all initial states and all Boolean functions.

In conclusion, the results of the ambiguity analysis of the integrative perturbation experiments show that both knock-down and over-expression techniques must be used for perturbation to reduce the ambiguity in the results of the logic-based GRN inference. Consequently, relying on the results of only knock-down experiments, as occurs in most of the studies in the literature, has a severe impact on the accurate predictability of the GRN models. By using only knock-down perturbations, we cannot achieve the unambiguous and certain identification of the GRNs. Moreover, if the data obtained by DO perturbations are integrated with the data of other omics layers, e.g., the genomics and proteomics layers, we can achieve a more accurate model of the GRN as a predictor of the cell behavior. This accuracy can be improved even more by employing multi-step perturbation experiments which is equivalent to multi-perturbations at a time.

### Experimental results

**Fig 11:**
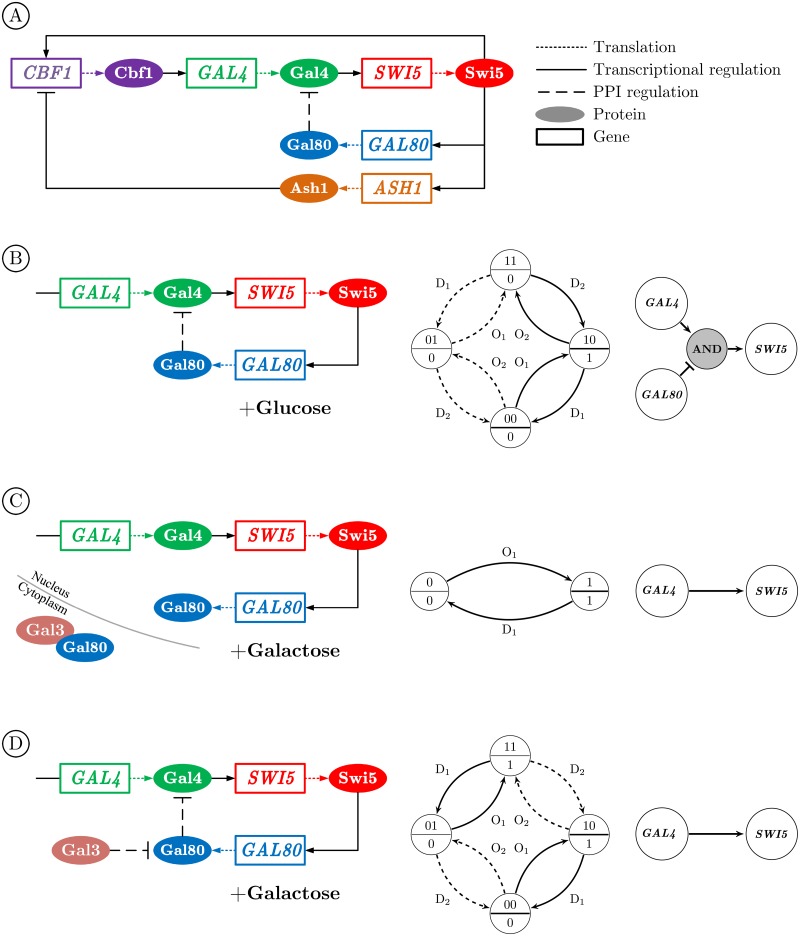
Identification of the underlying Boolean function of the regulatory effects of *GAL4* and *GAL80* on *SWI5* in a synthetic network in Yeast. A: The full schematic diagram of the synthetic network. B: The regulatory interactions in the absence of galactose (culturing cells in glucose) and the corresponding SPSD and the identified Boolean function, i.e., (*GAL4*)AND($\bar{GAL80}$). In the SPSD, RF_1_ and RF_2_ refer to *GAL4* and *GAL80*, respectively. C-D: The regulatory interactions in the presence of galactose in the cases of the dissociation and the non-dissociation model, respectively. In both cases of C and D, the identified Boolean function only reflects the activatory effect of *GAL4* on *SWI5*, i.e., *GAL4*→*SWI5*, despite the existing PPI between Gal4 and Gal80 in D.

To demonstrate the impact of applying different types of perturbation and integrating multi-omics data on identifying the Boolean functions in logic-based model of the GRNs, we analyze the real dataset of a synthetic network of five genes regulating each other through a variety of regulatory interactions in the yeast *Saccharomyces cerevisiae* (Fig 11A) [29]. The time series and steady-state expression data after multiple perturbations are measured by using quantitative real-time RT-PCR (q-PCR). The network can be switched on or off by culturing cells in galactose or glucose, respectively. To investigate the effect of different types of multi-omics data, we focus only on the regulatory interactions of *GAL4* and *GAL80* on *SWI5* in Fig 11A. In this network, the produced protein Swi5 acts as the activator of *GAL80* gene.

In the synthetic network, the promoter of gene *SWI5* is the *GAL1-10* promoter, which is regulated by galactose via the Gal4 transcription factor. In the absence of galactose, or the presence of glucose here, Gal80 protein binds to Gal4 acting as a repressor and prevents the expression of *SWI5* by Gal4 (Fig 11B). However, in the presence of galactose, Gal4 is free of the inhibitory effect of Gal80 and activates the transcription of *SWI5*. There are two models for the interaction of Gal4 and Gal80 in the presence of galactose [30]. In the dissociation model, Gal80 dissociates from Gal4 and interacts with Gal3 in the cytoplasm (Fig 11C). Hence, the nuclear concentration of Gal80 reduces due to the interaction between Gal3 and Gal80 proteins, and consequently, Gal4 can initiate the transcription of *SWI5*. In this model, we expect to not observe any PPI between Gal4 and Gal80. In the second model, i.e., non-dissociation model, a tripartite complex of Gal4, Gal80 and Gal3 exists in the nucleus that the PPI between Gal3 and Gal80 prevents the inhibitory effect of Gal80 on Gal4 (Fig 11D). In this model, we expect to observe PPI between Gal80 and Gal4. Note that we have no PPI information in this dataset, but based on the above biological knowledge and the presence or the absence of galactose, we presume that the PPI between Gal4 and Gal80 can be observed or not.

Different types of perturbations on *GAL4* and *GAL80* in two cases of the presence (+Galactose) and the absence (+Glucose) of galactose are listed in Table 4. These perturbations cover all combinations of the input states; hence, it is expected to identify the underlying Boolean function of the interactions uniquely. The Boolean function is identified in two cases of +Galactose and +Glucose separately. In the first step, the information about the promoter of *SWI5*, which is our *cis*-element data, provides evidence that *GAL4* is the regulator of *SWI5*. However, *cis*-element data cannot help us to recognize *GAL80* as an RF because it regulates *SWI5* indirectly by PPI. In the second step, we use the PPI data to recognize other potential RFs of *SWI5*. The PPI between Gal4 and Gal80 proteins could be observed in two cases of +Glucose (Fig 11B) and the non-dissociation model of +Galactose (Fig 11D). Hence, *GAL80* could be considered as a putative regulator of *SWI5* in these cases. *GAL4* remains the only regulator of *SWI5* in the dissociation model of +Galactose (Fig 11C).

**Table 4:** The list of perturbations on *GAL4* and *GAL80* to study their regulatory effects on *SWI5* in the presence of galactose and glucose.

		+Galactose		+Glucose	
*GAL4*	*GAL80*	*SWI5*	Perturbation	*SWI5*	Perturbation
**0**	**0**	0	Direct perturbation is not available (the result of +Glucose is adopted)	0	Shifting cells from galactose to glucose (time series data)
**0**	**1**	0	Over-expression of *GAL80* Down regulates all other genes Excess of Gal80 binds and represses Gal4, even in the presence of galactose	0	Over-expression of *GAL80* Down regulates all other genes
**1**	**0**	1	Over-expression of *GAL4* while *GAL80* is inactive	1	Over-expression of *GAL4* while *GAL80* is inactive
**1**	**1**	1	Shifting cells from glucose to galactose (time series data)	0	Direct perturbation is not available PPI between Gal4 and Gal80 proteins in glucose turns off *SWI5*

Both time series and steady state expression data are utilized to cover all state combinations of *GAL4* and *GAL80*. There is not any perturbation in this dataset for the two input states of 00 in galactose (+Galactose) and 11 in glucose (+Glucose). Regarding the input state 00 in galactose, we adopted the results of +Glucose experiment because other perturbations show that Gal4 and Gal80 act as the activator and inhibitor of *SWI5*, respectively. Therefore, lack of both of them should result in down regulation of the target gene. For the case of the input state 11 in glucose, we use our biological knowledge to determine the state of *SWI5*, i.e., the absence of galactose results in switching off the target gene. Moreover, if we observed PPI between Gal4 and Gal80 proteins in glucose, it reveals that both *GAL4* and *GAL80* genes are expressed, i.e., the input state is 11.

The SPSDs of the above cases and the corresponding Boolean functions are shown in Fig 11. Fig 11B shows that *SWI5* is regulated by the Boolean function (*GAL4*)AND($\bar{GAL80}$) in the absence of galactose. In the case of the dissociation model of +Galactose in Fig 11C, the only RF of *SWI5* is *GAL4* and the SPSD shows that it regulates as an activator, i.e., *GAL4*→*SWI5*. In the case of the non-dissociation model of +Galactose shown in Fig 11D, although we have evidence for the regulatory effect of both *GAL4* and *GAL80* on *SWI5*, the ultimate Boolean function obtained by the SPSD reflects only the effect of *GAL4* on *SWI5* as an activator, i.e., *GAL4*→*SWI5*. In other words, in the presence of galactose, Gal80 cannot prevent Gal4 from activating the expression of *SWI5*; hence, it does not play any role in the prediction of the state of *SWI5*. Indeed, although we have two RFs in the SPSD of Fig 11D, we are in one of the cases shown in Table 1 that the Boolean function has only one input variable. In summary, in the presence of galactose, *GAL80* does not influence the expression of *SWI5*, regardless of the model used for the interactions between Gal4 and Gal80 proteins.

## Conclusion

The logic-based models of the gene regulatory networks (GRNs), i.e., Boolean GRNs, are required in order to predict the cellular behavior in different circumstances. However, these models encounter restrictions and difficulties with the visibility of all gene regulatory interactions as well as the unambiguous identification of the underlying Boolean functions in the network. The main goal of this paper was to investigate and analyze these restrictions and difficulties. To overcome these restrictions, the effects of integrating different omics layers and employing different types of perturbations, i.e., the knock-down and the over-expression techniques, as well as applying single and multiple perturbations were studied here.

In this paper, we presented an optimistic analysis obtaining the upper bounds on the performance of the modeling in terms of visibility and ambiguity. First of all, we observed in this analysis that utilizing both knock-down and over-expression techniques, as the perturbation in order to identify the underlying Boolean functions, was required to achieve a better performance in the modeling. Furthermore, to achieve the best performance, we had to employ multi-perturbations, or equivalently, the multi-step single perturbations proposed in this work. The use of the mono-type and single perturbation experiments, like the knock-down experiments, usually observed in the literature due to the financial and the practical restrictions, resulted in missing and ambiguous interactions in our modeling. Moreover, the outcome of the analysis revealed that integrating more omics layers in the GRN inference resulted in fewer missing interactions and less ambiguity in the model. By integrating all transcriptomic, *cis*-element, and PPI data and combining them with multiple knock-down and over-expression perturbations, we obtained the least ambiguous model of the GRN. In terms of visibility, utilizing the *cis*-element and PPI data played the most important role in reducing the number of the missing interactions in the network. Finally, increasing the number of the regulators of a target gene made the task of the logic-based modeling of the GRN severely challenging.

In reality, the expected ambiguity in the GRN model will be greater than the results of our optimistic analysis because of either practical restrictions and difficulties, such as the missing data and noise, or not applying some of our assumptions in the analysis. For instance, any protein interacting with a regulatory factor of a target gene is not necessarily itself a regulator, but we considered it optimistically as a putative regulator. Therefore, this increase of the ambiguity observed in the real situations makes us treat the results of the studies not including various omics layers and perturbation types and not performing enough multiple perturbations with extreme caution.

The main assumption in our optimistic analysis was that all PPI and *cis*-element information are perfectly known. However, with the current technologies and databases, such complete information is far from being available in the immediate future. For example, one of the main sources of known and predicted PPIs is the STRING (Search Tool for the Retrieval of Interacting Genes/Proteins) database [31]. According to the latest snapshot of statistics for STRING, announced in May 2017 for version 10.5 [32], it contains 1,380,838,440 interactions of 9,643,763 proteins from 2031 organisms. However, all these interactions are not reliable, and in fact, only 2%, 5%, and 23% of them are with the highest, high, and medium or better confidence level, respectively. In addition, some ambiguities exist inherently in data that makes reaching to the complete knowledge impossible. For example, in prokaryotes, *cis*-elements are long enough to be alone sufficient for determining the corresponding TFs uniquely. However, in eukaryotes having a larger genome, the DNA motifs are too short to define the genomic position of the TF’s targets uniquely [33]. Therefore, we will have ambiguity and overlap in matching between the TFs and the existing *cis*-elements beside the target genes. In these cases, the second-order properties of the promoter regions can play a role in transcriptional regulation, requiring integration of other kinds of omics data.

The outcome of this paper constitutes the object of our future work. In the next step, we will develop a method for experiment design based on our proposed integrative multi-step perturbation experiment. In this method, according to the results of the first step of the perturbations, the most probable networks matching with our data will be determined, and then, the next required steps of the perturbation to identify the Boolean network with the minimum ambiguity and the least number of perturbations will be suggested. Finally, we want to develop an online tool to help researchers design time- and cost-efficient experiments.

## Supporting information

S1 Fig The main structure of the SPSD for *k* = 4. For clarity, only D perturbations are depicted. In the case of the DO perturbation, there are two transition edges, i.e., D_*i*_ and O_*i*_, in the reverse direction. To show the SPSD in a 2-dimensional representation, some state numbers (gray nodes) are depicted more than once.
(PDF)

S1 Table The values of *P_v_* and *P_fv_* obtained in the visibility analysis. (PDF)

S2 Table The values of *P_fv,s_* in the cases of DO and DO+PPI. In the case of DO+cis(+PPI), *P_fv,s_* is 1 for all values of *s*.
(PDF)

S3 Table The average number of the Boolean functions having a common set of transitions over all Boolean functions and all initial states for single D and DO perturbations. (PDF)

S4 Table The values of *P_ua_* obtained in the ambiguity analysis. (PDF)
