# Supplementary material for: Ambiguity in logic-based models of gene regulatory networks: An integrative multi-perturbation analysis
Source: PLoS One. 2018 Nov 20;13(11):e0206976. doi: 10.1371/journal.pone.0206976 (PMC6245684; doi:10.1371/journal.pone.0206976)
Supplement: S1 Fig — For clarity, only D perturbations are depicted. In the case of the DO perturbation, there are two transition edges, i.e., Di and Oi, in the reverse direction. To show the SPSD in a 2-dimensional representation, some state numbers (gray nodes) are depicted more than once. (PDF) [file pone.0206976.s001.pdf]

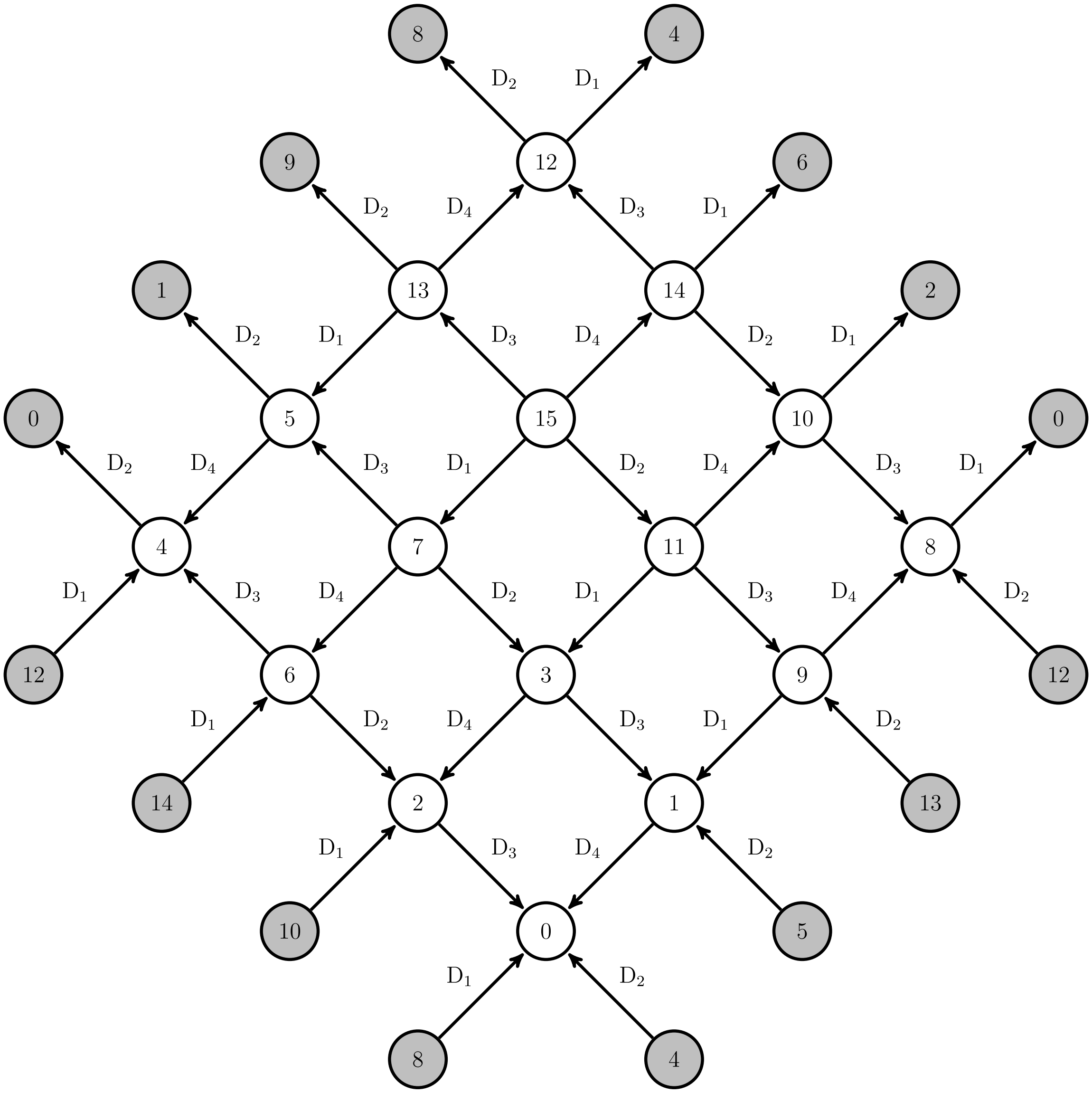

S1 Fig. The main structure of the SPSD for $k = 4$. For clarity, only D perturbations are depicted. In the case of the DO perturbation, there are two transition edges, i.e., D_i and O_i, in the reverse direction. To show the SPSD in a 2-dimensional representation, some state numbers (gray nodes) are depicted more than once.
