# Supplementary material for: Ambiguity in logic-based models of gene regulatory networks: An integrative multi-perturbation analysis
Source: PLoS One. 2018 Nov 20;13(11):e0206976. doi: 10.1371/journal.pone.0206976 (PMC6245684; doi:10.1371/journal.pone.0206976)
Supplement: S1 Table — (PDF) [file pone.0206976.s002.pdf]

**S1 Table. The values of $P_v$ and $P_{fv}$ obtained in the visibility analysis.**

| | $P_v$ | | | $P_{fv}$ | | |
|---|---|---|---|---|---|---|
| | $k$=2 | $k$=3 | $k$=4 | $k$=2 | $k$=3 | $k$=4 |
| **D** | 0.60 | 0.52 | 0.50 | 0.10 | 0.02 | 0.004 |
| **D+PPI** | 0.70 | 0.74 | 0.79 | 0.20 | 0.11 | 0.06 |
| **DO** | 0.60 | 0.52 | 0.50 | 0.40 | 0.15 | 0.06 |
| **DO+PPI** | 0.65 | 0.66 | 0.69 | 0.50 | 0.38 | 0.30 |
| **+cis(+PPI)** | 1 | 1 | 1 | 1 | 1 | 1 |
