# Supplementary material for: Ambiguity in logic-based models of gene regulatory networks: An integrative multi-perturbation analysis
Source: PLoS One. 2018 Nov 20;13(11):e0206976. doi: 10.1371/journal.pone.0206976 (PMC6245684; doi:10.1371/journal.pone.0206976)
Supplement: S2 Table — In the case of DO+cis(+PPI), Pfv,s is 1 for all values of s. (PDF) [file pone.0206976.s003.pdf]

S2 Table. The values of $P_{fv,s}$ in the cases of DO and DO+PPI. In the case of DO+cis(+PPI), $P_{fv,s}$ is 1 for all values of s.

	s	**0**	**1**	**2**	**3**	**4**	**5**	**6**	**7**	**8**	**9**	**10**	**11**	**12**	**13**	**14**	**15**
k=4	**DO**	0.06															
	**DO+PPI**	0.06	0.06	0.06	0.25	0.06	0.25	0.25	0.50	0.06	0.25	0.25	0.50	0.25	0.50	0.50	0.94
k=3	**DO**	0.15															
	**DO+PPI**	0.15	0.15	0.15	0.52	0.15	0.52	0.52	0.89								
k=2	**DO**	0.4															
	**DO+PPI**	0.4	0.4	0.4	0.8												
