# Supplementary material for: Ambiguity in logic-based models of gene regulatory networks: An integrative multi-perturbation analysis
Source: PLoS One. 2018 Nov 20;13(11):e0206976. doi: 10.1371/journal.pone.0206976 (PMC6245684; doi:10.1371/journal.pone.0206976)
Supplement: S3 Table — (PDF) [file pone.0206976.s004.pdf]

S3 Table. The average number of the Boolean functions having a common set of transitions over all Boolean functions and all initial states for single D and DO perturbations.

	$k = 2$			$k = 3$			$k = 4$		
	**D, DO**	**+PPI**	**+cis(+PPI)**	**D, DO**	**+PPI**	**+cis(+PPI)**	**D, DO**	**+PPI**	**+cis(+PPI)**
**Single D**	2	1.5	2.2	16	13.9	37.1	2048	2020.3	8747.3
**Single DO**	2	1.8	1.4	16	14.5	13.7	2048	2028.2	2018.7
