# Supplementary material for: Ambiguity in logic-based models of gene regulatory networks: An integrative multi-perturbation analysis
Source: PLoS One. 2018 Nov 20;13(11):e0206976. doi: 10.1371/journal.pone.0206976 (PMC6245684; doi:10.1371/journal.pone.0206976)
Supplement: S4 Table — (PDF) [file pone.0206976.s005.pdf]

S4 Table. The values of P_{ua} obtained in the ambiguity analysis.

	**$k = 2$**			**$k = 3$**			**$k = 4$**		
	**D, DO**	**+PPI**	**+cis(+PPI)**	**D, DO**	**+PPI**	**+cis(+PPI)**	**D, DO**	**+PPI**	**+cis(+PPI)**
**1D**	0.05	0.15	0.45	0.001	0.01	0.03	1.9E-6	2.9E-5	1.6E-4
**2D**	0.1	0.2	0.5	0.005	0.04	0.08	1.5E-5	2.4E-4	6.1E-4
**3D**	-	-	-	0.009	0.08	0.12	6.2E-5	9.61E-4	1.6E-3
**4D**	-	-	-	-	-	-	1.2E-4	1.9E-3	2.7E-3
**1DO**	0.2	0.3	0.8	0.009	0.03	0.07	3.1E-5	1.5E-4	4.9E-4
**2DO**	0.4	0.5	**1**	0.04	0.12	0.33	2.5E-4	1.2E-3	0.004
**3DO**	-	-	-	0.07	0.25	0.71	0.001	0.005	0.016
**4DO**	-	-	-	0.15	0.38	**1**	0.004	0.02	0.07
